# Appearance of claudin-5^+^ leukocyte subtypes in the blood and CNS during progression of EAE

**DOI:** 10.1186/s12974-021-02328-3

**Published:** 2021-12-21

**Authors:** Dylan Krajewski, Debayon Paul, Shujun Ge, Evan Jellison, Joel S. Pachter

**Affiliations:** 1 Blood-Brain Barrier Laboratory, UConn Health, 263 Farmington Ave., Farmington, CT 06030 USA; 2 Department of Immunology, UConn Health, 263 Farmington Ave., Farmington, CT 06030 USA; 3 PureTech Health, 6 Tide Street, Boston, MA 02210 USA

**Keywords:** Tight junctions, Leukocytes, Neuroinflammation, Blood–brain barrier

## Abstract

**Background:**

Tight junctions (TJs) are membrane specializations characteristic of barrier-forming membranes, which function to seal the aqueous pathway between endothelial cells or epithelial cells and, thereby, obstruct intercellular solute and cellular movement. However, previous work from our laboratory found that claudin-5 (CLN-5), a TJ protein prominent at the blood–brain barrier (BBB), was also detected, ectopically, on leukocytes (CLN-5^+^) in the blood and central nervous system (CNS) of mice with experimental autoimmune encephalomyelitis (EAE), a neuroinflammatory, demyelinating disease that is a model for multiple sclerosis. CLN-5 was further shown to be transferred from endothelial cells to circulating leukocytes during disease, prompting consideration this action is coupled to leukocyte transendothelial migration (TEM) into the CNS by fostering transient interactions between corresponding leukocyte and endothelial junctional proteins at the BBB.

**Methods:**

To begin clarifying the significance of CLN-5^+^ leukocytes, flow cytometry was used to determine their appearance in the blood and CNS during EAE.

**Results:**

Flow cytometric analysis revealed CLN-5^+^ populations among CD4 and CD8 T cells, B cells, monocytes and neutrophils, and these appeared with varying kinetics and to different extents in both blood and CNS. CLN-5 levels on circulating T cells further correlated highly with activation state. And, the percentage of CLN-5^+^ cells among each of the subtypes analyzed was considerably higher in CNS tissue than in blood, consistent with the interpretation that CLN-5^+^ leukocytes gain preferred access to the CNS.

**Conclusion:**

Several leukocyte subtypes variably acquire CLN-5 in blood before they enter the CNS, an event that may represent a novel mechanism to guide leukocytes to sites for paracellular diapedesis across the BBB.

**Supplementary Information:**

The online version contains supplementary material available at 10.1186/s12974-021-02328-3.

## Introduction

Tight junctions (TJs) are membrane specializations that promote adhesion between both adjacent epithelial cells and endothelial cells. Integral transmembrane proteins of TJs, including the respective claudin (CLN) and junctional adhesion molecule families, in addition to occludin, span the aqueous, intercellular space as fibrils to interact with their counterparts on the opposing cells [1] In this capacity, they form a barrier of high electrical resistance that severely restricts diffusion of fluids and solutes [2]. It has further been argued that TJs also pose an impediment to leukocyte migration across vascular and other tissue boundaries [3–6]. Alterations in the expression and/or localization of TJ proteins, in response to inflammatory and/or toxic stimuli, often results in edema, infiltration of pathologic soluble and cellular blood components, and a variety of local and systemic diseases [1–7].

While TJ proteins are most commonly and prominently expressed by epithelial or endothelial cells, whereby they perform barrier-type functions, ‘ectopic’ expression by several migratory cell types, notably leukocytes, has been reported [8–13]. Yet despite growing awareness of their occurrence, the significance of leukocyte-associated TJ proteins in many of these instances remains enigmatic. One possibility is that they participate in leukocyte transendothelial migration (TEM) via a “zipper mechanism”, whereby endothelial–endothelial junctional contacts are temporarily replaced with homophilic or heterophilic interactions between corresponding leukocyte and endothelial junctional proteins [14–16]. Analogously, junctional adhesion molecules of intra-epithelial lymphocytes have been proposed to interact with their epithelial junctional protein neighbors in the small intestine [10]. Supporting such an interactive role, our group [17] detected CLN-5-expressing (CLN-5^+^) leukocytes in blood and among perivascular infiltrates in spinal cord of mice with experimental autoimmune encephalomyelitis (EAE), a neuroinflammatory, demyelinating disease that serves as a model of multiple sclerosis (MS) [18]. These CLN-5^+^ leukocytes were observed as early as the pre-clinical phase of EAE. Moreover, at least some of this CLN-5 was transferred from endothelial cells to leukocytes. There has also been report of CLN-5^+^ leukocytes in blood of MS patients, and that presence of these cells is upregulated during disease relapse while downregulated following a positive response to anti-inflammatory therapy [19]. As CLN-5 is a major TJ protein of the microvascular endothelium comprising the blood–brain barrier (BBB) and a crucial determinant of BBB status [20], these results could suggest CLN-5 appearance on leukocytes plays a key role in leukocyte TEM into the central nervous system (CNS) in the course of neuroinflammation. In this regard, CLN-5^+^ leukocytes might serve a pioneering function, penetrating the TJs of the BBB earlier in the inflammatory process and, thereby, clearing the way for other leukocytes to follow.

However, to begin clarifying the significance of CLN-5^+^ leukocytes during neuroinflammation, several questions regarding their appearance need to be addressed. What leukocyte subtypes bear this tight junction protein? When do they appear in the blood and CNS during disease? Does the amount of CLN-5 on individual leukocytes change as disease evolves? And, is the acquisition of CLN-5 associated with leukocyte activation or inflammatory state? To this end, mice were induced to develop EAE, and CLN-5^+^ leukocytes from blood and CNS evaluated by robust flow cytometry and correlation analysis at varying times during pre-clinical and clinical stages of disease to identify leukocyte characteristics associated with CLN-5 positivity. Results suggest acquisition of CLN-5^+^ status is not indiscriminate, but more likely associated with particular leukocytes at certain times and events in the inflammatory process, and could facilitate the ability of cells to cross the BBB into the CNS early in disease.

## Methods

### Animals

Female C57BL/6J mice (Charles River Laboratories), age 8–10 weeks, were used throughout. All mice were maintained under specific pathogen-free conditions and all animal protocols were in compliance with Animal Care and Use Guidelines of UConn Health (Animal Welfare Assurance # A3471-01).

### EAE induction

Active EAE was induced as described [17, 21]. Subcutaneous flank injection of 300 μg MOG_35-55_ peptide (MEVGWYRSPFSRVVHLYRNGK, Millipore Sigma) in complete Freund’s adjuvant (CFA, Difco) containing 300 μg *Mycobacterium tuberculosis* was performed on day 0 (D0), and supplemented by intraperitoneal injections of 500 ng pertussis toxin (PTX, List Biological) on D0 and D2 to heighten the autoimmune reaction to MOG peptide [22, 23].

Blood and CNS (brain + spinal cord) tissues were harvested at different days (D) post EAE induction for detection and immunophenotype analysis of CLN-5^+^ leukocytes in the peripheral and central compartments at various stages of disease: D0 (naïve, no disease), D6 and D9 (early and late pre-clinical disease, respectively), D12 (initiation of clinical disease), and D15 (mid-acute disease). Additionally, groups of mice receiving only CFA and Ptx were analyzed at D6 and D12, and served as controls for any possible effects due solely to adjuvants. A total of 6 mice were used per condition.

### Isolation of circulating and CNS-infiltrating leukocytes

Total peripheral blood leukocytes (PBLs) and CNS-infiltrating leukocytes were isolated from each mouse and analyzed separately. To obtain PBLs, anti-coagulated blood was collected from the thoracic cavity following transcardiac perfusion with PBS containing heparin (10 units/ml). PBLs were subsequently purified from the collected blood by passing them through an Acrodisc® White Blood Cell filter (PALL Labs). The trapped leukocytes were extracted from the filter with PBS and centrifuged at 1500 rpm for 5 min at 4 ºC. The cell pellet was then resuspended in cell staining buffer (Biolegend) for flow cytometry.

**Fig. 1:**
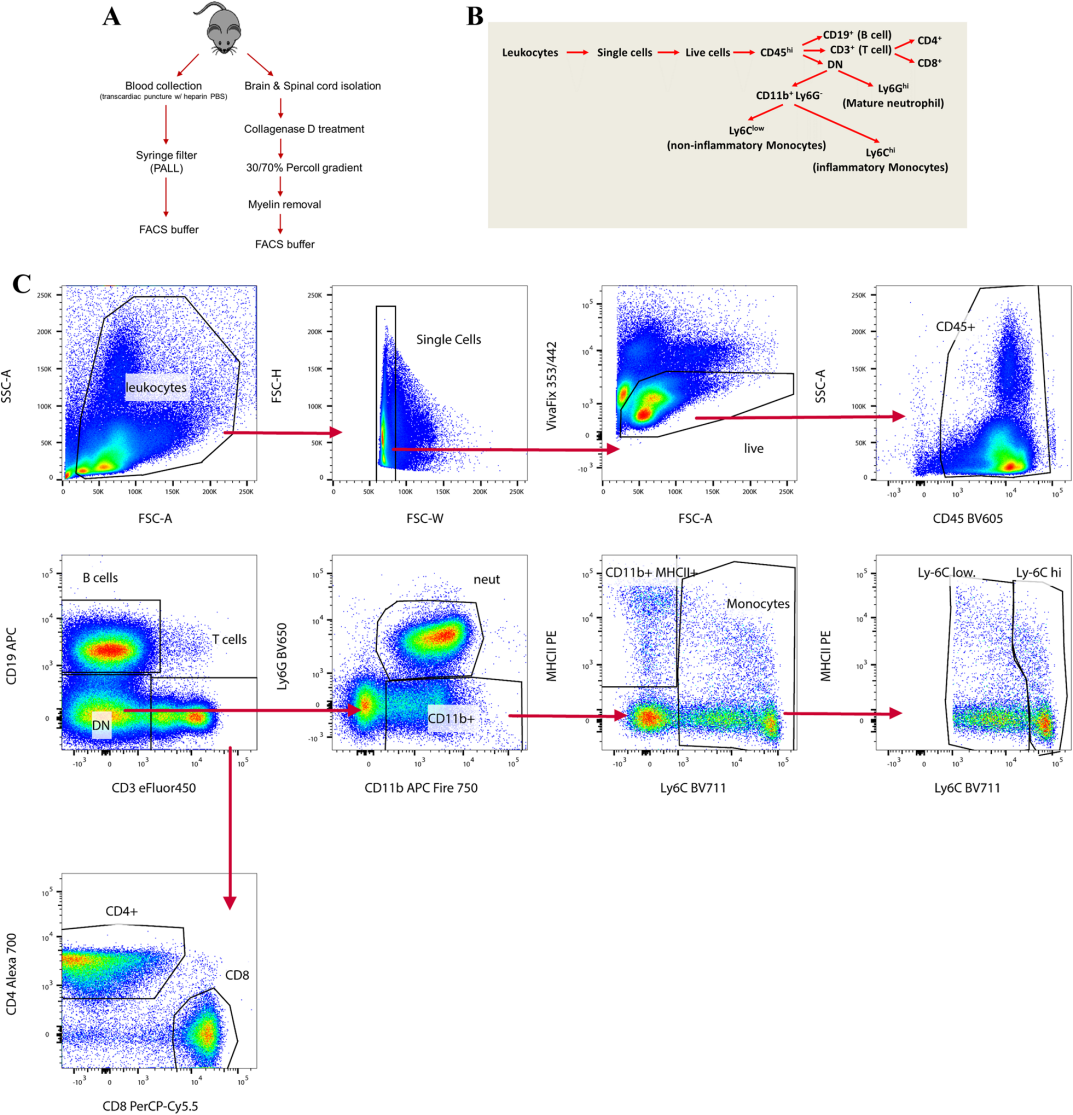
Schemes for leukocyte analysis. **A** Experimental design for separate isolation of leukocytes from peripheral blood and CNS (brain + spinal cord) for flow cytometry. **B** Scheme for identification of leukocyte subtypes. **C** Gating strategy for immunophenotyping of leukocytes

CNS-infiltrating leukocytes were isolated by combined enzymatic digestion/density gradient centrifugation largely as described [24], with minor modifications. Immediately following perfusion and collection of blood, brain and spinal cord were isolated from each mouse and transferred, together, to 4 ml ice-cold RPMI in a 35-mm cell culture dish. The combined CNS tissues from individual mice were minced using a 15 T scalpel blade (Feather Blades, World Precision Instruments), and dispersed using a disposable tissue grinder tube (Fisher Scientific). Subsequently, CNS tissue was digested with collagenase D (Roche Diagnostics) and DNase I (Sigma-Aldrich) as detailed by Anstadt et al. [24], and then resuspended in 10 ml of RPMI media containing 10% fetal bovine serum (Gibco BRL) to neutralize enzyme activities. CNS tissue was then passed through a 70-µm filter (BD) to remove particulates, and centrifuged at 1500 rpm for 5 min at 4 ºC. The pellet was resuspended in 70% Percoll (Sigma-Aldrich). A 30% Percoll solution was first aliquoted in a 15 ml tube, and gently underlaid with the 70% Percoll solution containing the resuspended CNS cells. The gradient was spun at 500×*g* for 20 min at 22 ºC. Myelin debris was removed from the top, and leukocytes collected from the interface. Leukocytes were resuspended in 9 ml RPMI and centrifuged at 1500 rpm for 5 min at 4ºC. Pelleted cells were resuspended in cell staining buffer (Biolegend) in preparation for identifying CLN-5^+^ cells and immunophenotyping by flow cytometry. Figure 1A, B shows the schemes for leukocyte isolation and immunophenotyping.

### Flow cytometry

Leukocytes isolated from blood and CNS tissue of each mouse were separately treated with F_c_ block (BD Bioscience) and incubated with the phenotyping antibodies (Additional file 1: Table S1) against CD45, CD4, CD8a, CD11b, CD11c, CD19, CD44, Ly6-C, and Ly6-G (Biolegend), CD3 (eBioscience), and MHCII (Abcam) at 4 ºC for 20 min. Antibodies with non-overlapping fluors were selected using FluoroFinder 2.0. Cells were stained with VivaFix 353/422 (BioRad) to exclude dead cells from analysis. The stained cells were washed with cell staining buffer (Biolegend), and treated with cell fixation and permeabilization kit reagents (LifeTech). The permeabilized cells were post-fixed with 2% paraformaldehyde, and then incubated with Alexa® 488-conjugated CLN-5 antibody (LifeTech) at 4 ºC overnight. Stained cells were analyzed on a LSR II (Becton Dickinson) using the gating strategy described in Fig. 1C, and data analysis was performed with FlowJo software version 10.7 (BD/Treestar). Absolute numbers of CLN-5^+^ cells of each leukocyte subtype were determined using precision count beads (Biolegend) according to the manufacturer’s protocol. Briefly, equal volumes of beads and sample were combined and mixed well prior to analysis. Counting beads were gated to separate them from cells based on forward angle light scatter (FSC) versus APC fluorescence, as the beads alone gave a brighter signal than the CD19^+^ B cell population. The event number for each cell population was then divided by the bead count for that sample and multiplied by the lot-specific bead concentration to obtain a readout of cells/uL. To account for differences in detector sensitivity across experimental timepoints, an FMO control was prepared for CLN-5-Alexa488 on each day, as this was the main target for quantification. The CLN-5 MFI for each mouse was then divided by the FMO during analysis to create the final standardized value.

### Statistical analysis

Numerical data are displayed as the mean ± SEM. All groups were compared using a one-way ANOVA with a Tukey’s multiple comparisons post hoc test. A cutoff of *P* < 0.05 was used for significance. All bar graphs and statistical calculations were created using Prism version 9 (Graphpad).

To determine the relationship between CLN-5 abundance and leukocyte activation/inflammatory state, linear regression analysis was performed comparing single cell fluorescence intensity values for CLN-5 to those for the respective activation markers. Specifically, CLN-5 was compared to CD44 for T cells, CD18 for neutrophils, and Ly-6C for monocytes. Only leukocytes from blood were subject to this analysis, as these were reasoned to represent the broadest spectrum of activation, leukocytes having migrated into the CNS considered to be already functionally activated [25, 26]. First, raw fluorescence intensity values for each cell within a given leukocyte subset were exported from FlowJo V10, and the data set downsized by using a random numbers generator within Microsoft Excel for Office 365 (version 2103) to non-biasedly select 10,000 events (each representing an individual cell), from each sample. Since the vast majority of the T cells were only minimally activated, to avoid this population dominating or obscuring the relationship between CLN-5 abundance and increasing activation, the range of activation marker fluorescence intensity was divided into thirds, sorting the 10,000 selected cells into low, medium or high activation/inflammatory states. Due to a relatively low number of cells in the highest activation category, all cells in this group were included in the regression analysis, and an equal number selected from the low and medium categories after again sorting each category with a random numbers generator. This approach was employed separately for all six mice at each time-point, and all mice were combined for the final regression analysis. From this data set Pearson correlation coefficients were calculated, and significance was determined.

Additional tests for the relationship between CLN-5 abundance and activation state were performed on T cells, as it is generally accepted EAE is a T cell-mediated disease [27, 28], and there is evidence T cells can penetrate TJs at a time when the BBB is intact [29]. Specifically, a second activation marker, CD11c [30], was used in combination with CD44 to determine this relationship. Linear mixed effects models (R Core Team version 4.0.5) were used to model CLN-5, including the fixed effects of CD44 or CD11c only, and both CD44 and CD11c, each with a random intercept per mouse to account for the dependence of cells within mice. The effects of CD44 and/or CD11c were estimated and tested using t-tests under the linear mixed effects model and the pseudo-R^2^ was reported. A 3-D interpolation was also employed to find the line of best fit to estimate the value of CLN-5 fluorescence intensity from that of CD44 or CD11c within the XLfit (version 5.5) add-on for Microsoft Excel.

## Results

### Appearance of CLN-5^+^ leukocytes in blood and CNS during EAE

**Fig. 2:**
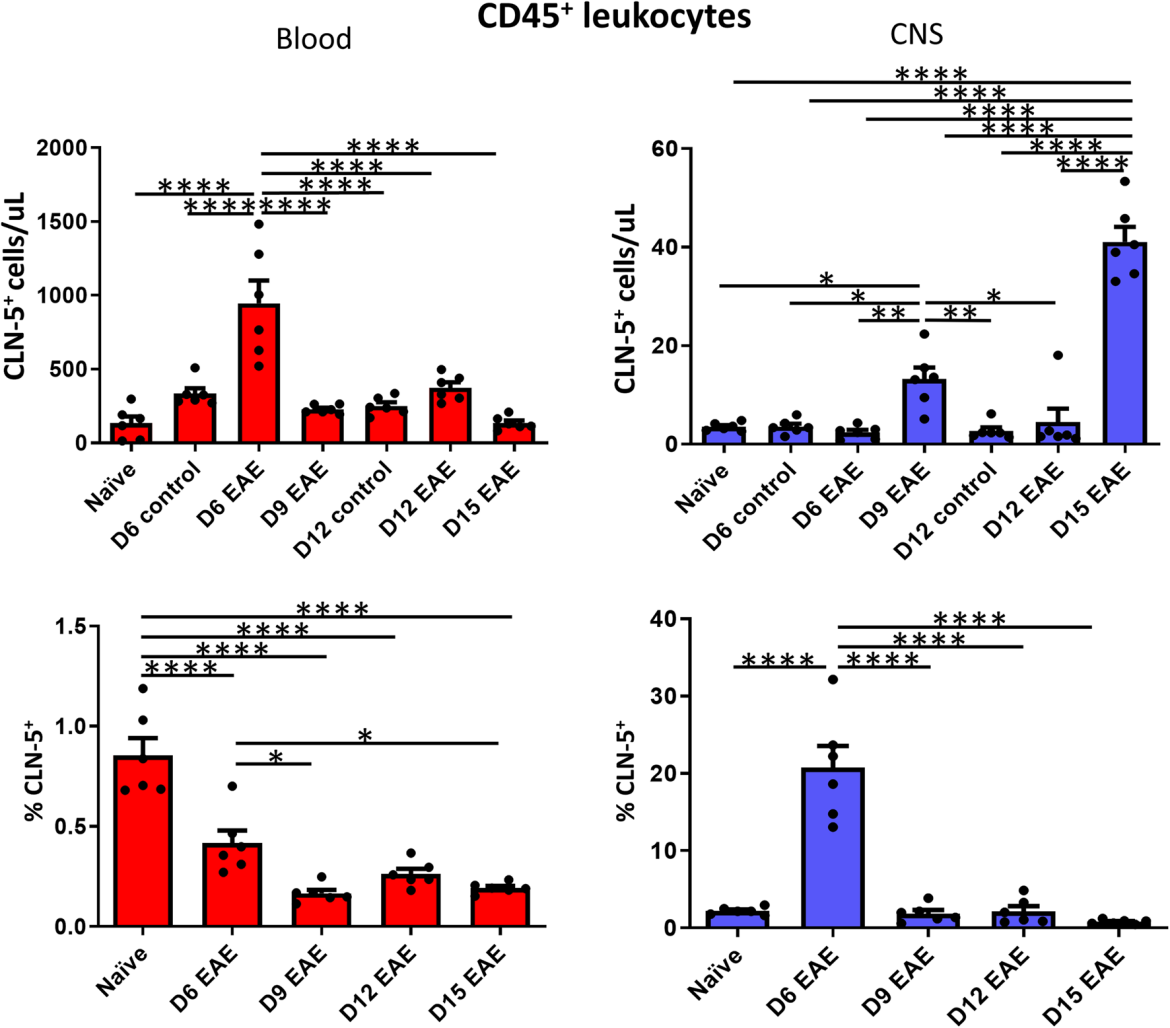
Appearance of CLN-5^+^ CD45^+^ cells in blood/CNS during EAE. At the indicated days (D) following EAE induction, peripheral blood and CNS (brain + spinal cord) leukocytes were separately collected from individual mice, and analyzed by flow cytometry to identify CLN-5^+^ CD45^+^ cells. Number of CLN-5^+^ leukocytes and their percentage of the CD45^+^ population in blood/CNS are reported. Individual data points are overlaid for each group. Naïve mice received no injections, and control mice on D6 and D12 received adjuvants alone. Each experimental group contained six mice. Data are expressed as mean + SEM. Bars indicate comparison between groups. *p < 0.05; **p < 0.01; ***p < 0.001; ****p < 0.0001

To get a sense of how disease progression was accompanied by appearance of CLN-5-bearing leukocytes, the *total* number of CD45^+^CLN-5^+^ cells in both blood and CNS compartments was determined, as was their percent representation in each tissue (Fig. 2). Representative flow plots from which these data were derived are further presented in Additional files 2, 3, 4, 5, 6, 7, 8: Figs. S1–S7. Brain and spinal cord were processed together to yield total CNS leukocytes, and flow cytometry performed to identify the CLN-5^+^ cells.

The amount of CD45^+^CLN-5^+^ cells in blood peaked early in the disease course, showing a dramatic increase at D6, the earliest time-point measured, then falling sharply at D9 to a value near that of naïve mice for the remainder of disease. The situation was reversed in the CNS, with the peak number CD45^+^CLN-5^+^ cells in the time course detected at D15 EAE. This delay in appearance of CD45^+^CLN-5^+^ cells in the CNS is consistent with the transit of CLN-5^+^ leukocytes from the circulation into the brain and spinal cord during the neuroinflammation that accompanies EAE. That control animals receiving adjuvant alone did not show similar elevation of CD45^+^CLN-5^+^ cells—behaving more like naïve animals—argues the appearance of these cells is specifically linked to MOG-induced disease.

The percent of CD45^+^ cells in blood that were CLN-5^+^ was highest in naïve mice, dropping steeply as early as D6. After D6, the percent CLN-5^+^ cells decreased yet more by D9, remaining at this low level until D15. Notably, CLN-5^+^ cells never reached, on average, more than 0.4% of the CD45^+^ cells in blood during disease. The situation in CNS was vastly different. The percent of CD45^+^ cells in CNS that were CLN-5^+^ showed a dramatic peak early in disease at D6. This level then precipitously declined by D9, and remained near constant until D15. Of further distinction is that 20% of CD45^+^ cells in CNS were CLN-5^+^ at D6, 50 × the value seen in blood at this time-point. Such a vastly disproportionate accumulation of CLN-5^+^ cells in the CNS is consistent with a preference for CLN-5^+^ rather than CLN-5^−^ leukocytes to extravasate from the blood and enter the brain and/or spinal cord early in EAE pathogenesis.

### Contribution of leukocyte subtypes to the CLN-5^+^ population

After confirming the EAE-associated wave of CLN-5^+^ leukocytes in the blood, and its subsequent advance into the CNS, the next question was: is bearing CLN-5 a general leukocyte property during EAE, or do particular subtypes show a preference for this action? Utilizing qualitative immunofluorescence microscopy, Mandel et al. [19] described CLN-5 staining mainly on B cells, T cells and monocytes from normal human blood, and further detailed by qRT-PCR and Western blotting upregulation of CLN-5 RNA and protein, respectively, in total blood leukocytes from MS patients experiencing relapse. To see if there were any parallels with human disease, we analyzed these leukocyte subtypes in blood and CNS by flow cytometry at the various EAE time-points. Additionally, neutrophils were also evaluated for comparison. All the different leukocytes examined showed some segment of their respective populations to be CL5^+^ by flow cytometry, though the timing of their appearance and relative contributions varied by both subtype and tissue compartment.

#### *B cells*

**Fig. 3:**
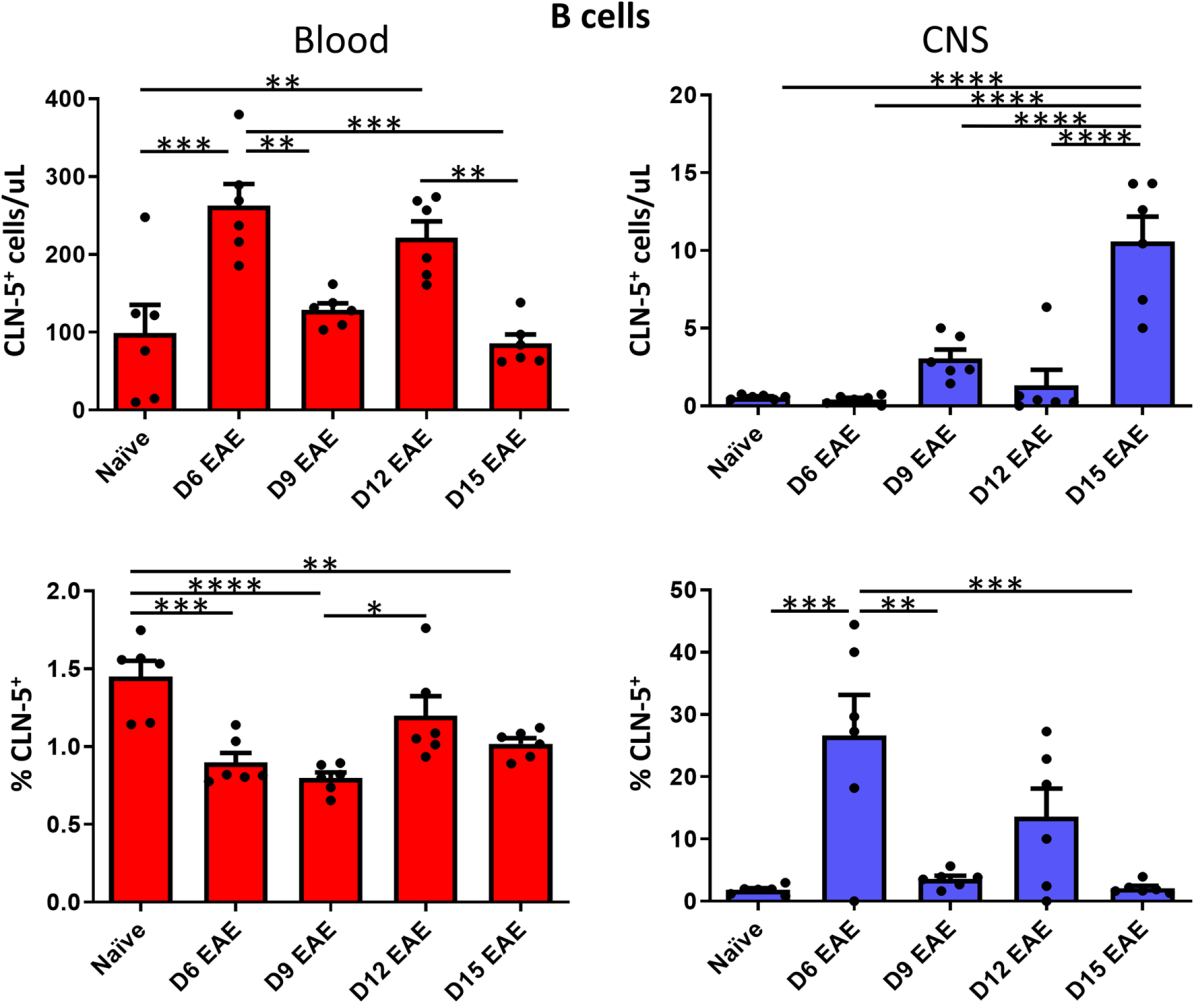
Appearance of CLN-5^+^ B cells in blood/CNS during EAE. Peripheral blood and CNS leukocytes were collected at various days (D) following EAE induction, and analyzed by flow cytometry to identify CLN-5^+^ CD19^+^ B cells. Number of CLN-5^+^ B cells and their percentage of the B cell population in blood/CNS are reported. Individual data points are overlaid for each group. Each experimental group contained six mice. Data are expressed as mean + SEM. Bars indicate comparison between groups. *p < 0.05; **p < 0.01; ***p < 0.001; ****p < 0.0001

CLN-5^+^ B cells were detected in both blood and CNS compartments (Fig. 3). The number of CLN-5^+^ B cells peaked in the blood early, at D6. Given the direction of leukocyte movement from blood-to-CNS, the peak number of CLN-5^+^ B cells in the CNS expectedly occurred later, with the greatest number seen at D15. In blood, the percent CLN-5^+^ cells remained nearly constant, fluctuating around 1%. In CNS, the percent CLN-5^+^ cells peaked early on D6 at ~ 25%, then decreased. As was the case for total CD45^+^ cells, the proportion of B cells that were CLN-5^+^ was much greater in the CNS than in the blood, possibly reflecting a predisposition for CLN-5^+^ versus CLN-5^−^ B cells to extravasate into parenchymal brain and/or spinal cord tissue. It might further reveal that CLN-5^+^ B cells are the *pioneering* B cells that first enter the CNS, structurally clearing the way for CLN-5^−^ B cells to follow. The continued extravasation of CLN-5^+^ cells among a far greater number of CLN-5^−^ B cells would still result in expansion of the CLN-5^+^ B cell population in the CNS but at a reduced proportion. Alternatively, some deposition of CLN-5 protein onto the surface of initially CLN-5^−^ cells might occur during the midst of paracellular diapedesis, as this process has been reported to be accompanied by disruption and remodeling of TJ proteins [31, 32].

#### *T cells*

**Fig. 4:**
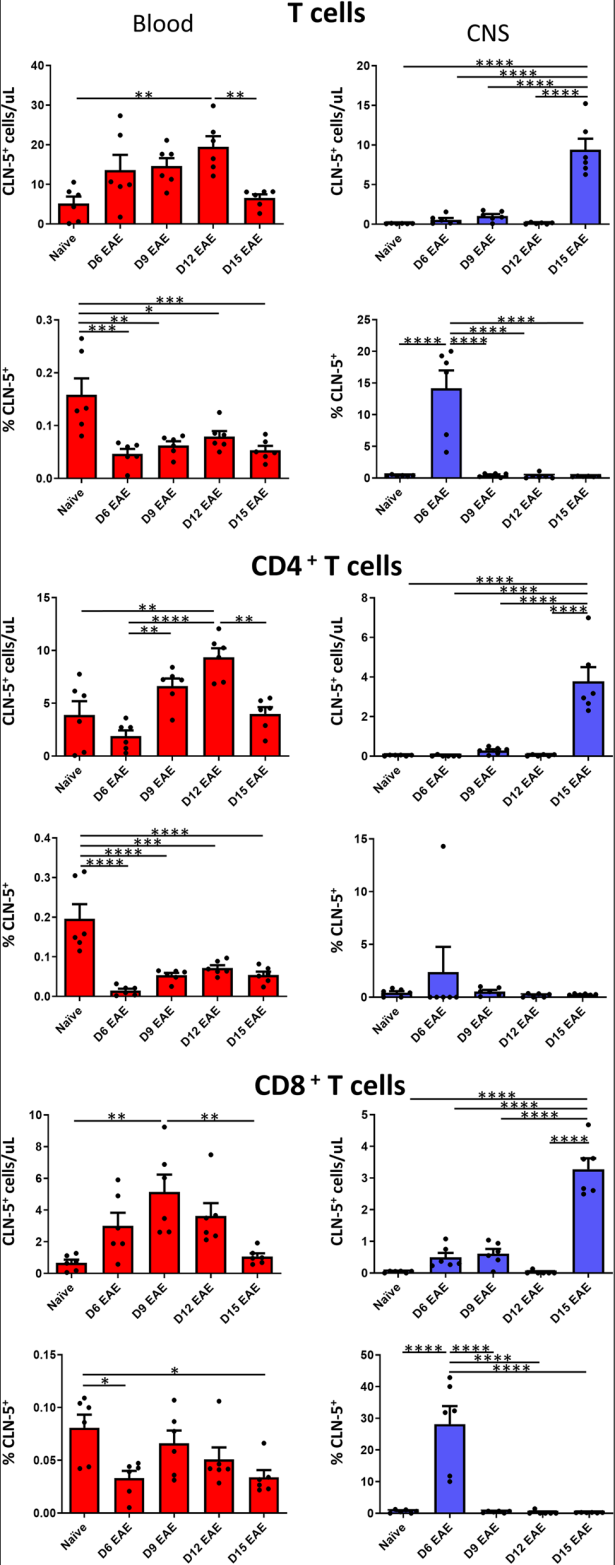
Appearance of CLN-5^+^ T cells in blood/CNS during EAE. Peripheral blood and CNS leukocytes were collected at various days (D) following EAE induction, and analyzed by flow cytometry to identify CLN-5^+^ cells among total T cells, CD4^+^ T cells, and CD8^+^ T cells. Number of CLN-5^+^ T cells of each subtype and their percentage of each population in blood/CNS are reported. Individual data points are overlaid for each group. Each experimental group contained six mice. Data are expressed as mean + SEM. Bars indicate comparison between groups. *p < 0.05; **p < 0.01; ***p < 0.001; ****p < 0.0001

CLN-5^+^ T cells (CD3^+^) were also observed in both blood and CNS (Fig. 4). However, the pattern of appearance of CLN-5^+^ T cells in blood was more delayed than that for B cells, peaking later at D12. Their appearance in CNS mirrored that for B cells, being maximal at D15—after peak appearance in blood—again consistent with movement of CLN-5^+^ cells from blood-to-brain/spinal cord. The percent of T cells in blood that were CLN-5^+^ showed no overt difference throughout the time course of disease—varying only slightly from 0.04% to 0.08%—but, unexpectedly, was highest in naïve mice, and then dropped precipitously during EAE. This level, moreover, never surpassed more than 0.08% at any time-point during disease—far below the nearly 1.0% level observed for B cells. In contrast to the situation in blood, the percent of CLN-5^+^ T cells in the CNS did show a peak at D6, as was also the case for B cells. Likewise, the percent CLN-5^+^ T cells in CNS at this time-point, ~ 14%, was 280× that found in blood—again perhaps reflecting a propensity for CLN-5^+^ leukocytes—across varied subtypes—to enter the CNS first, leading the way for the preponderance of CLN-5^−^ cells to follow.

The CLN-5^+^ T cell population was further resolved into CD4 and CD8 subtypes, as both contribute variably to CNS inflammation during EAE [33–37]. The appearance of CLN-5^+^ CD4 cells in both blood and CNS virtually mirrored that observed for total T cells, peaking in the former at D12, and in the latter at D15. CLN-5^+^ CD8 cells were only somewhat different, with the highest level of cells detected in blood at D9. CLN-5^+^ CD8 cells, like their CD4 counterparts, were observed to be at the highest level in the CNS at D15, once again, trailing the peak seen in the blood. The percent of both CLN-5^+^ CD4 and CD8 cells in blood showed no drastic fluctuations throughout the time course—though CD8 cells showed the biggest difference between D6 and D9—and each of the two T cell subtypes showed a drop during disease from that seen in naïve mice. The percent of both CLN-5^+^ CD4 and CD8 cells in CNS was highest at D6, though the value for CD4 cells was much lower and showed high variability. It is thus likely that CD8 cells, rather than CD4 cells, contributed more to the spike in percent total T cells in CNS being CLN-5^+^.

#### Monocytes

**Fig. 5:**
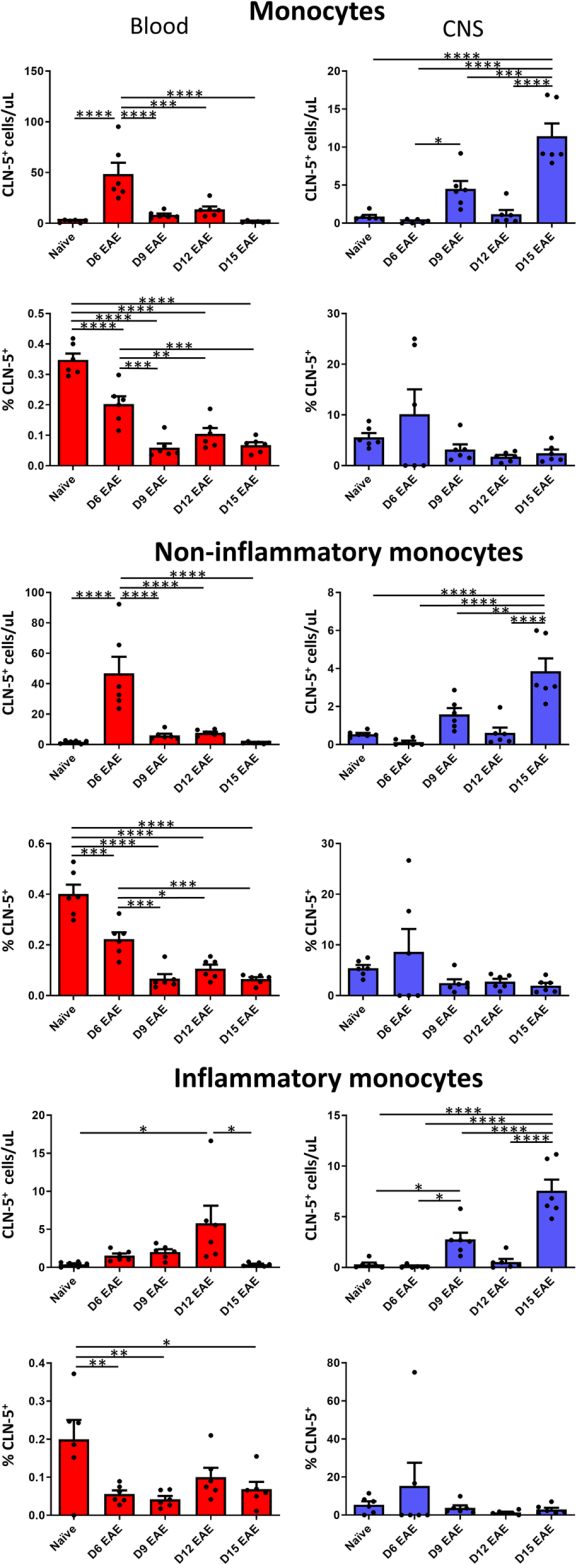
Appearance of CLN-5^+^ monocytes in blood/CNS during EAE. Peripheral blood and CNS leukocytes were collected at various days (D) following EAE induction, and analyzed by flow cytometry to identify CLN-5^+^ cells among total monocytes, non-inflammatory monocytes (Ly6C^lo^), and inflammatory monocytes (Ly6C^hi^). Number of CLN-5^+^ monocytes of each subtype and their percentage of each population in blood/CNS are reported. Individual data points are overlaid for each group. Each experimental group contained six mice. Data are expressed as mean + SEM. Bars indicate comparison between groups. *p < 0.05; **p < 0.01; ***p < 0.001; ****p < 0.0001

Like B cells, monocytes showed a peak number in blood at the earliest time-point, D6, while falling to a low at the last time-point, D15 (Fig. 5). And they too showed the inverse in the CNS, being maximal at D15, and minimum at D6. The percent of blood monocytes that were CLN-5^+^ was highest in naïve mice (0.35%), as was the case with T and B cells, but then gradually dropped at D6, and then fell even more at D9, from there on remaining near constant to D15 (0.1%). The proportional representation of CLN-5^+^ monocytes in blood was thus lower than that for B cells but higher than that for T cells. Fluctuations in the percent CLN-5^+^ monocytes in the CNS showed a similar scenario to that seen in blood, being highest at D6 (10%), then dropping at D9 and continuing near invariant through D15 (~ 2.5%). But, as with B cells, T cells and CD45^+^ cells in general, the maximum percent CLN-5^+^ monocytes in CNS was higher than that in blood by more than one order of magnitude, revealing a disproportionate association of CLN-5 with monocyte penetration of the BBB.

CLN-5^+^ monocytes were further sorted into two functional subsets characterized by distinct migratory and inflammatory properties: Ly6C^lo^ *non-inflammatory* monocytes, which display CXCR3-mediated recruitment and serve in a vascular patrolling capacity [38]; and Ly6C^hi^ *inflammatory* monocytes, which rapidly migrate into inflamed tissues in a CCR2-dependent manner [39], produce inflammatory cytokines [40], and are associated with an earlier onset and increased severity of EAE [41]. Separation in this way was meant to see if acquisition of CLN-5^+^ could be related to inflammatory status. In blood, the two monocyte subsets showed notably different appearances of CLN-5^+^ cells. The number of non-inflammatory monocytes peaked early at D6, then dropped sharply at D9 to near naïve level, and returned to naïve level by D15. In contrast, inflammatory monocytes gradually accumulated in blood during the course of EAE, steadily rising from the level found in naïve mice to a peak at D12, and then returning to normal by D15. Yet, despite their very dissimilar scenarios in blood, the two monocyte subsets displayed comparable appearances of CLN-5^+^ cells in CNS, both showing a small spike at D9, a brief return to normal, and then a peak at D15. The percentages of CLN-5^+^ cells among the respective non-inflammatory and inflammatory monocyte populations in blood were also comparable in both chronology and degree, being maximal in naïve animals (0.4% and 0.2%, respectively), dropping to < half these values at D6 EAE, and then hovering around a near constant value (< 0.1%) through D15. Likewise, non-inflammatory and inflammatory CLN-5^+^ monocytes displayed similar frequencies in CNS during EAE. Specifically, the percentages of CLN-5^+^ cells among both monocyte populations were slightly higher than naïve levels at D6 (ranging from ~ 8.6% [non-inflammatory]—15% [inflammatory]), dropping more than half in each case by D9, and then remaining at ~ these levels until D15. As with total monocytes, the peak percentages of both non-inflammatory and inflammatory monocytes in CNS were much greater than those found in blood—though there was considerable disparity between the two populations. The peak percentage of non-inflammatory monocytes in CNS was ~ 37.5× that for blood, while that for inflammatory monocytes was ~ 300× the corresponding blood value. A possible inference for this distinction may be that inflammatory monocytes are more dependent on CLN-5 for BBB transit than are non-inflammatory monocytes.

#### Neutrophils

**Fig. 6:**
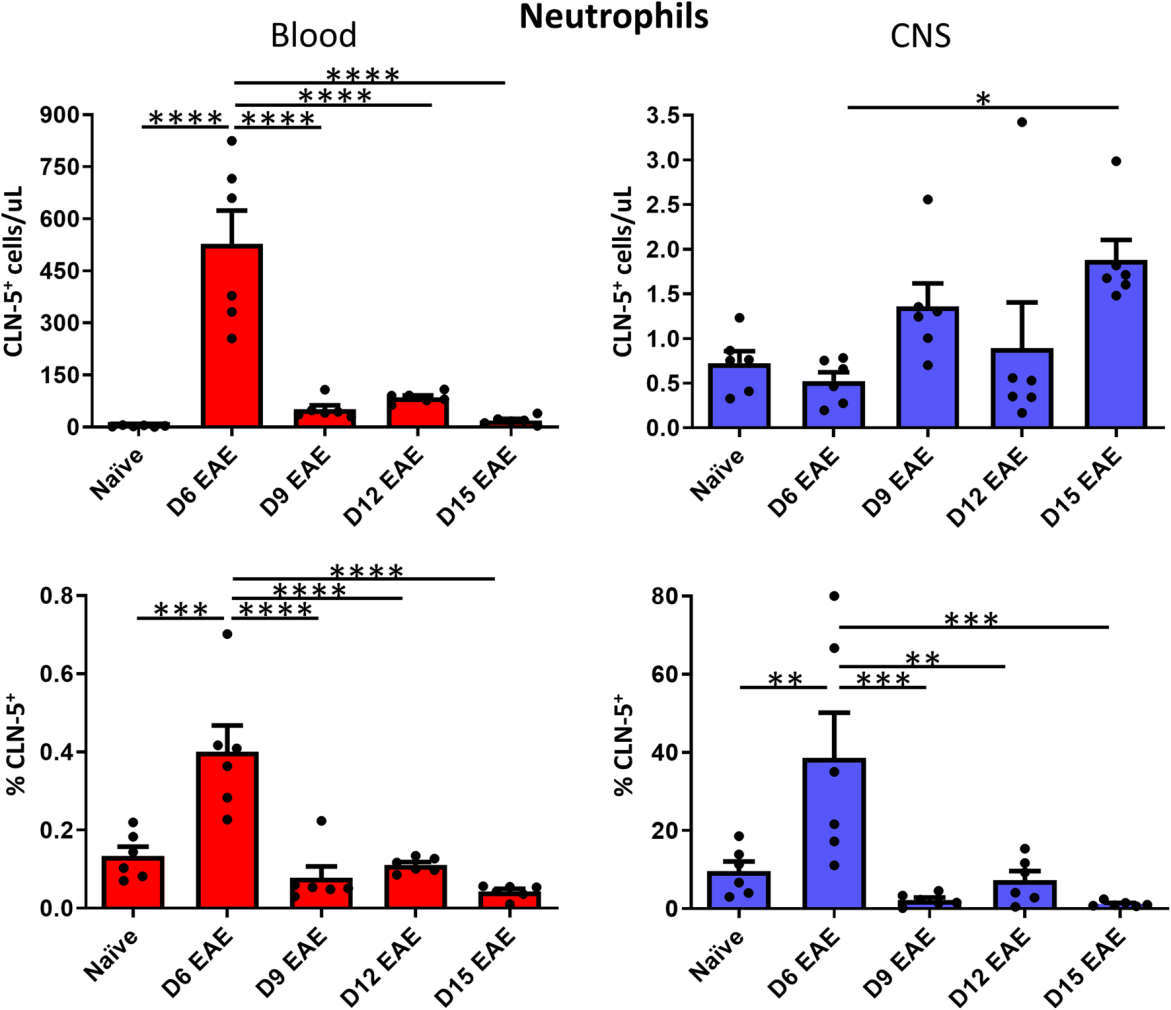
Appearance of CLN-5^+^ neutrophils in blood/CNS during EAE. Peripheral blood and CNS leukocytes were collected at various days (D) following EAE induction, and analyzed by flow cytometry to identify CLN-5^+^ neutrophils (Ly6G^hi^). Number of CLN-5^+^ neutrophils and their percentage of the neutrophil population in blood/CNS are reported. Individual data points are overlaid for each group. Each experimental group contained six mice. Data are expressed as mean + SEM. Bars indicate comparison between groups. *p < 0.05; **p < 0.01; ***p < 0.001; ****p < 0.0001

Though Mandel et al. [19] did not report neutrophils as being a prominent leukocyte subtype bearing CLN-5 protein in blood of healthy humans or those with MS, these cells were examined here as they enter the CNS and contribute to pathogenicity during EAE [42, 43] (Fig. 6). As with B cells and monocytes, the respective time courses of number of neutrophils in blood versus CNS were mirror images of each other, being highest in the blood at D6 and lowest at D15, while being lowest in the CNS at D6 and reaching a peak at D15. Similar to that found with B and T cells, the percent of CLN-5^+^ neutrophils in CNS was clearly highest at D6 (40%) and much greater (100×) than the percent detected in blood (at most, 0.4%), which likewise peaked at this early time. Of all the leukocyte subtypes, neutrophils achieved the highest percent CLN-5^+^.

### Leukocyte CLN-5^+^ mean fluorescence intensity (MFI) varies during EAE

**Fig. 7:**
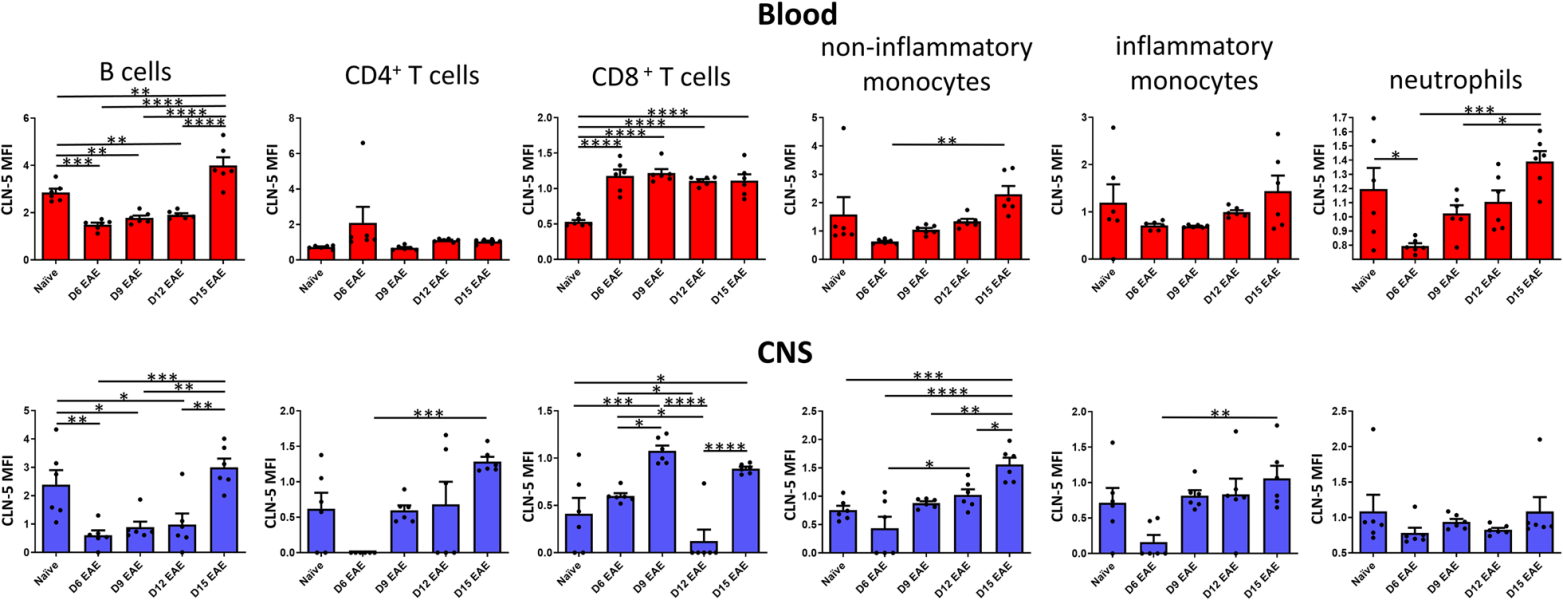
Relative amount of CLN-5 in leukocyte subtypes in blood and CNS during EAE. Peripheral blood and CNS leukocytes were collected at various days (D) following EAE induction, and analyzed by flow cytometry to yield normalized, CLN-5^+^ MFI values for the various leukocyte subtypes. Data are expressed as mean + SEM. Individual data points are overlaid for each group. Each experimental group contained six mice. Bars indicate comparison between groups. *p < 0.05; **p < 0.01; ***p < 0.001; ****p < 0.0001

In addition to changes in the total and relative number of CLN-5^+^ leukocytes in blood and CNS, the average, relative amount of CLN-5 protein per cell—reflected by normalized, CLN-5^+^ MFI values detected by flow cytometry—appeared to fluctuate as well. As all subtypes evaluated contributed to the overall CLN-5^+^ leukocyte population in blood and CNS, MFIs were determined for each one (Fig. 7).

#### B cells

After an initial drop from the level in naïve animals, CLN-5^+^ MFI in both blood and CNS trended upward gradually from D6 to D12, then peaked sharply at D15. As at or around this time of disease there is heightened inflammation, when TJs are being broken down and considerable CLN-5 protein is lost from the CNS vasculature [21, 44, 45], B cells may have acquired some CLN-5 protein that was liberated into the circulation, the inter-endothelial cleft, and/or perivascular space through proteolysis or other means [46, 47].

#### *T cells*

The two T cell subtypes displayed CLN-5^+^ MFI patterns that were each distinct from that of B cells, as well as from each other. CLN-5^+^ CD4 cells had the highest MFI in blood at D6, while the MFI of CLN-5^+^ CD8 cells remained invariant in blood during the time course of disease. The MFI patterns also differed in the CNS. MFI for CLN-5^+^ CD4 cells peaked at D15—the same time when most CD4 cells appeared in this compartment. By contrast, CLN-5^+^ CD8 cells showed the highest MFI value earlier, at D9—prior to their greatest accumulation in the CNS. Peak accumulation of CLN-5^+^ CD4 and CD8 cells in CNS thus occurred at the same time, or after, they achieved their maximal CLN-5^+^ MFI. Notably, save for one instance (CD4 T cells in blood at D6), MFI values during disease were > to those of naïve animals in both blood and CNS compartments, indicating T cells acquired additional CLN-5 protein in the course of EAE.

#### *Monocytes*

Unlike the different MFI patterns seen between the two T cell subsets, those for the respective monocyte subsets were similar in blood and CNS. For both subsets in both tissues, there was an increase in MFI as acute EAE evolved, with the steepest rise seen in non-inflammatory monocytes. This more robust change in MFI in non-inflammatory monocytes may, again, reflect that CLN-5 acquisition is more essential to disease-related activities in this population.

#### Neutrophils

The pattern of MFI for CLN-5^+^ neutrophils resembled that for B cells and monocytes, trending highest in both blood and CNS at D15.

### CLN-5^+^ intensity is related to leukocyte activation

**Fig. 8:**
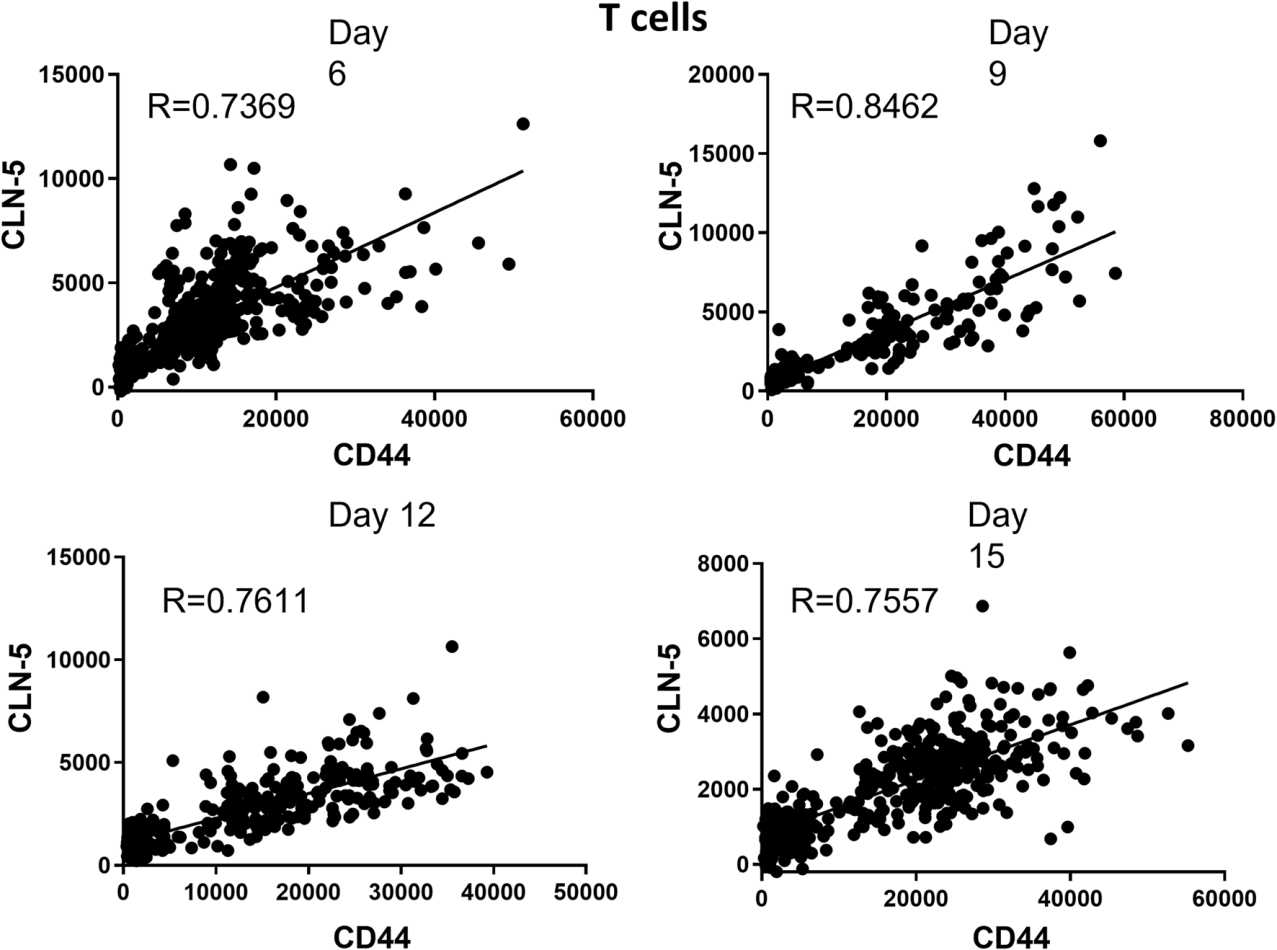
CLN-5 abundance correlates with activation state of T cells in blood. Peripheral blood leukocytes were collected at various days (D) following EAE induction, and analyzed by flow cytometry to yield raw fluorescence intensity values for CLN-5 and CD44 in individual T cells of each mouse leukocyte sample. Data were culled from 6 mice at each time-point to yield the regression lines and Pearson Product–Moment Correlation Coefficient (*r*)

Independent lines of evidence suggest there may be some connection between leukocyte acquisition of CLN-5 and level of activation. The migration of T cells across the BBB and entry into the CNS is dependent on these leukocytes being activated and expressing the necessary repertoire of adhesion molecules [48–50], a requirement that might also exist for other leukocyte subtypes [25]. And, Mandel et al. [19] reported that activation of human T cells in vitro resulted in upregulation of claudin-1, a member of the same TJ protein family as CLN-5. In light of these relationships, and our finding that, during EAE, CLN-5^+^ leukocytes appear in the CNS in greater proportion to their presence in blood for all subtypes examined, we further sought to determine if CLN-5 abundance in T cells, monocytes and neutrophils correlated with these leukocytes’ state of activation. To gauge this relationship, CLN-5 fluorescence intensity of individual cells, as determined by flow cytometry, was used as a surrogate measurement for CLN-5 abundance. Likewise, single cell-associated, fluorescence intensity of well-recognized activation markers was used for the respective leukocyte subtypes. The activation marker used for T cells was CD44, as it is upregulated in antigen-primed T cells and considered a key activation marker in EAE [51, 52]. For monocytes, though no activation markers, per se, were employed, the relationship between CLN-5^+^ intensity and inflammatory status, the latter judged by Ly6C intensity, was investigated. Lastly, CD18 was used to identify activated neutrophils, as it is required for extravasation [53], and its blockade shown to resolve EAE [54]. A Pearson Product–Moment Correlation Coefficient (*r*) was determined between intensity values for each of these markers and those of CLN-5^+^. Correlation analysis was confined to blood, as T cells that have entered the CNS are nearly all activated (Additional file 9: Fig. S8), though only a fraction are CLN-5^+^. The population of all leukocytes examined in blood, by contrast, displayed a broader spectrum of both activation states and CLN-5 intensities. For T cells, *r* showed remarkable consistency, ranging from 0.74 to 0.81 across all the time-points, being maximal at D9 and reflecting a strong linear association between CLN-5^+^ intensity and T cell activation status (Fig. 8).

To further reinforce this association, another marker for T cell activation, CD11c, was evaluated, as it is associated with high migratory potential [30] and correlated highly with CD44 expression (Additional file 10: Fig. S9). Linear mixed effects models revealed the relationship among intensities for CLN-5, CD44, and CD11c. Specifically, these reflected the percent variance in CLN-5 explained by CD44 and CD11c. The mean change in CLN-5 per unit change in CD44 was 0.82 (95% CI 0.76–0.88, *p* < 2e−16), reduced to 0.41 (95% CI 0.32–0.50, *p* = 3.06e−16), when adjusted for CD11c. Similarly, the mean change in CLN-5 per unit change in CD11c was 0.84 (95% CI 0.78–0.91, *p* < 2e−16), reduced to 0.51 (95% CI 0.41–0.60, *p* < 2e−16), when adjusted for CD44. The association of CD44 or CD11c with CLN-5 was significantly reduced when the other was included in the model as the two are highly correlated (Pearson correlation coefficient *r* = 0.81). Consistently, the percent of variance explained by CD44 and CD11c (pseudo-R-squared 0.75) did not significantly reduce by dropping one or the other (pseudo-R-squared 0.66 for CD44 only and 0.70 for CD11c only). A 3D interpolation and surface plot further revealed the linear relationship among CLN-5, CD44, and CD11c intensities, underscoring the connection between CLN-5 acquisition and T cell activation (Additional file 11: Fig. S10).

By contrast to the evident relationship between CLN-5 intensity and activation in T cells, the linear correlation found between monocyte CLN-5 intensity and inflammatory status was not as strong (Additional file 12: Fig. S11). Specifically, *r* values ranged from 0.038 to 0. 549 across the EAE time-points. This aligns with the concept that “activation” and “inflammation” are separate, measurable parameters of monocytes that could, but do not necessarily, overlap [55]. It also coincides with the observation that different monocyte subsets of varied inflammatory states found in the blood all infiltrate the CNS during EAE [56].

The situation for neutrophils reflected an even weaker relationship (Additional file 13: Fig. S12). There was no evidence of linear correlation between CLN-5^+^ intensity and neutrophil activation, the latter reflected by CD18 intensity, with *r* ranging only from 0.062 to 0.251 across the time course of disease. Notably, CD18 intensity for individual neutrophils varied over 25-fold, yet that for CLN-5 only fluctuated ~ twofold. This could indicate that the ability of neutrophils to augment their CLN-5 status—at least during EAE—is restricted when compared to that of T cells. In support of this possibility, the range of CLN-5 intensity in T cells is much greater than that in neutrophils for all but one time-point during EAE.

## Discussion

**Fig. 9:**
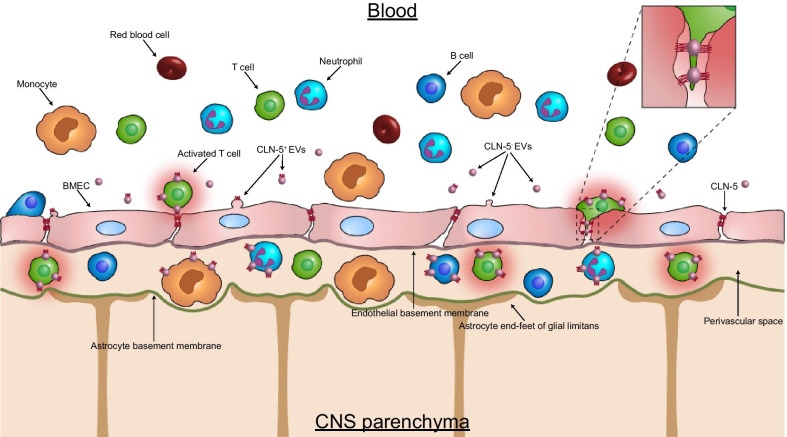
CLN5^+^ leukocyte subtypes in blood and CNS during EAE. Shown is a schematic of how acquisition of CLN-5 by leukocytes is envisioned to take place early in disease at the intact blood–brain barrier (BBB). Leukocytes in the blood are depicted focally acquiring CLN-5 from nascent CLN-5^+^ extracellular vesicles (EVs) released by brain microvascular endothelial cells (BMEC) of the BBB, though some endogenous gene expression of CLN-5 has been detected in leukocytes in normal and disease states [17, 19]. And, while populations of CLN-5^+^ cells are observed in all the leukocyte subtypes shown, T cells, specifically, exhibit a high correlation between CLN-5 abundance and activation state. Since activation is required for efficient T cell entry into the non-inflamed CNS [48–50], T cells crossing the intact BBB are depicted with a halo representing their high level of activation. Moreover, a higher proportion of leukocytes with attached CLN-5^+^ EVs is shown in the perivascular space compared to the blood, as current results reveal a higher percentage of CLN-5^+^ leukocytes in the CNS than in the circulation – possibly suggesting a preference for CLN-5^+^ leukocytes that are the most highly activated to extravasate. The inset depicts a T cell undergoing paracellular diapedesis by exploiting a hypothetical zipper mechanism whereby leukocyte-associated CLN-5^+^ EVs temporarily form bridges with CLN-5 on the BMEC surface

This laboratory previously reported that CD45^+^CLN-5^+^ cells, presumably leukocytes, appeared in the blood and CNS of C57BL/6 mice during EAE, as judged by high-resolution 3D immunofluorescence [17]. And, CLN-5^+^ status was further validated by Western blotting. The present studies used flow cytometry to confirm and extend this past work, identifying specific leukocyte subtypes and some of their derivative subsets that appeared in both tissues over the acute course of this neuroinflammatory disease, shedding some light on the significance of these cells (Fig. 9). What stands out is that CLN-5^+^ status is not random among leukocytes, instead being dependent upon leukocyte subtype/subset, disease state, and tissue site, among possibly other factors.

What mechanism(s) underlies the appearance of CLN-5^+^ leukocytes, and what significance does this population hold for the inflammatory disease process? A rudimentary hypothesis regarding the coupling of CLN-5 acquisition by leukocytes to their extravasation across CLN-5-bearing TJs of the BBB has previously been put forth by our group [17]. And, though empirical evidence in support of this hypothesis awaits detailed mechanistic studies that are beyond the scope of this report, some clues may be offered by the time course of appearance of these cells in the respective blood and CNS compartments, and their disproportionate representation in the two tissues. It is thus remarkable that, despite CLN-5^+^ status being cell, time, and tissue selective during EAE, the proportion of CLN-5^+^ cells in blood never exceeded much more than 0.4%. By contrast, 20% of total leukocytes in the CNS were CLN-5^+^ early in disease (D6). Such a discrepancy might indicate that CLN-5^+^ phenotype is only critical for few cells in blood, and those cells are enriched in the nascent influx of leukocytes into the CNS at the inception of neuroinflammation.

As to how CLN-5 comes to be present in leukocytes—an ectopic locale—Mandel et al. [19] detected protein and RNA in peripheral blood leukocytes (PBLs) from both normal and MS patients. Likewise, we observed both protein and gene expression in PBLs from naïve mice and those with EAE [17]. These findings are consistent with there being at least some endogenous CLN-5 expression by leukocytes. But, as this laboratory previously showed, a portion of leukocyte-associated CLN-5 is also obtained from an exogenous source. Specifically, using chimeras of WT bone marrow transplanted into transgenic mice expressing eGFP-CLN-5 under a Tie-2 promoter [17], our group demonstrated CLN-5^+^ status of leukocytes is partially conferred by endothelial-derived CLN-5, suggesting endothelial-to-leukocyte transfer.

Though the route of this transfer remains uncertain, it is unlikely to be entirely through passive contamination of leukocytes by endothelial CLN-5 liberated into the bloodstream, inter-endothelial cleft or perivascular space by proteolysis of TJs during TEM. Were this the case, circulating leukocytes would not be expected to acquire CLN-5 from endothelial cells during pre-clinical disease—as they have been shown to do [17]—when leukocyte extravasation into the CNS parenchyma is limited and the BBB still physically intact. It would appear, instead, that exogenous CLN-5 is provided to leukocytes when the BBB is still robust and endothelial supply of this protein is ample.

Several findings help form the basis for tying leukocyte-associated CLN-5 origin and function together. The following observations from the present studies hold particular significance for which, when, and where leukocytes gain CLN-5^+^ status: (1) CLN-5^+^ leukocytes of all major subtypes are found in both blood and CNS during EAE; (2) their peak appearance in blood precedes that in CNS; (3) there is significant correlation between the state of activation of T cells and CLN-5^+^ intensity; (4) the percentage of CLN-5^+^ cells of any subtype is disproportionately greater in the CNS compared to blood. And, results from previous studies bring both a source and vehicle for CLN-5 transfer into focus: (1) CLN-5 is transferred from endothelial cells to leukocytes during EAE [17]; and (2) extracellular vesicles (EVs) released by brain microvascular endothelial cells that comprise the BBB contain CLN-5 [17] as well as another TJ protein, occludin [57] and can bind to leukocytes in vitro [17].

A unifying hypothesis to relate all these observations is that, during neuroinflammation, EVs containing CLN-5 are released into the blood, which then bind to leukocytes and facilitate their migration across the BBB. Acquisition of CLN-5 in this manner is clearly not obligatory for leukocyte TEM, as CLN-5^−^ leukocytes are found in the CNS. Rather, CLN-5 may play a supplemental role, assisting with TEM by providing certain leukocytes navigational cues to direct them to the TJs of the inter-endothelial cleft, so they might then navigate the endothelium via a zipper mechanism. Such a scenario would be envisioned particularly for those leukocytes undergoing paracellular TEM; i.e., between adjacent endothelial cells, during which remodeling of TJs [32] could allow for the possibility of inter-endothelial TJ complexes to be transiently replaced by endothelial–leukocyte ones. For leukocytes extravasating by transcellular TEM; i.e., through the body of the endothelium, a process initiated by invasive podosomes and achieved by breaching of endothelial cells via a transcellular pore that leaves TJs morphologically intact [58–60], acquiring endothelial CLN-5 would appear to offer no advantages.

That all leukocyte subtypes investigated exhibited variable CLN-5^+^ status before and during EAE might impart that while the ability to display this TJ protein is universal among these blood cells, the extent they do so is cell, time, and context dependent. The latter two factors may relate to the degree of inflammation. In this regard, paracellular TEM of T cells has been proposed to be favored over the transcellular route at times and situations when the BBB is most intact [29]. While it may seem paradoxical that a leukocyte would prefer to cross where TJs are the strongest, endothelial-to-leukocyte transfer of CLN-5 and the zipper mechanism it conceivably supports, could be overriding determinants.

But, if CLN-5^+^ status is a determinant for directing paracellular TEM, then why do CLN-5^+^ leukocytes have very low representation in the peripheral blood even during EAE? And why would circulating leukocytes need to acquire more CLN-5 protein from endothelial cells, when these immune cells express mRNA for this TJ component in both the healthy and diseased state [19]? Again, these two features may be related. Basal, high-level expression of CLN-5 by most circulating leukocytes may be considered both unnecessary and detrimental. Since transfection of CLN genes ectopically into normally TJ-free cells causes aggregation [61], leukocytes expressing CLN-5 at too high a level might suffer a similar fate and impede hemodynamics. Thus, low-level, endogenous expression of CLN-5 by few leukocytes would both prevent these cells from aggregating in the blood, yet still potentially afford them homotypic binding sites for endothelial-derived CLN-5—possibly in the form of CLN-5^+^ EVs. This scenario could support CLN-5^+^-EVs serving as multi-dentate bridges, temporarily connecting leukocytes and endothelial cells at inter-endothelial locales where TJs abound. It would, therefore, seem most beneficial for only a small population of leukocytes to add to their CLN-5 repertoire by donation from a separate, normally TJ-rich, endothelial cell pool, and for this to happen under circumstances restricted in both space and time. Such focal transfer of exogenous, EV-associated CLN-5 from endothelial cells to leukocytes expressing endogenous CLN-5 might explain why the % CLN-5^+^ leukocytes in blood decreases during EAE from that found in naïve mice. Acquisition of exogenous CLN-5 by some circulating leukocytes would not immediately alter the % CLN-5^+^ leukocytes in blood, but could soon lead to their depletion due to heightened extravasation capacity.

A way to spatio-temporally restrict CLN-5 acquisition by leukocytes is for these cells to obtain this protein only when in close association with endothelial cells. This could explain the high correlation of CLN-5 intensity with T cell activation status, as activated CD4 T cells have been proposed to have a unique capacity to bind endothelial membranes [62] and intimate contact between T cells and endothelial cells can instruct the former to modify their level of activation [63, 64]. Contact between leukocytes and endothelial cells might also provide the signal for the latter to release CLN-5 in the form of CLN-5^+^ EVs. Indeed, in a situation bearing close parallels to that described here, activated CD4 T cells were found to bind “endothelial membrane fragments”—including those containing adhesion molecules CD31 (enriched at intercellular junctions [65, 66]) and CD62e—during TEM [62]. It has further been observed that memory T cells that have first undergone TEM across vascular endothelial cells show enhanced, subsequent migration across lymphatic endothelial cells [67]. Collectively, these reports reinforce the prospect of coordination of endothelial-to-leukocyte protein transfer with facilitation of diapedesis. The signal(s) that stimulate such protein transfer remain unknown, though it is intriguing to consider that some aspect of leukocyte binding to vascular endothelium is contributory. In this way, CLN-5^+^ released by endothelial cells at the apical surface—possibly in the form of EVs—might engage overhanging leukocytes in a juxtracrine manner, thereby avoiding dilution by the circulation and minimizing off-target effects.

The coupling of endothelial-to-leukocyte transfer of CLN-5 with leukocyte diapedesis might further explain the low representation of CLN-5^+^ leukocytes in blood, and their consistently higher representation in CNS—at times reaching more than 100-fold in excess for some leukocyte subtypes. Once the first cadre of invading leukocytes has exploited this process to disrupt inter-endothelial TJs, the endothelial CLN-5 available for transfer may become depleted, and/or the need for assistance in negotiating the paracellular pathway mitigated. It may take only few such pioneer leukocytes—by using acquired CLN-5 to effect the zipper mechanism of TEM—to leave the paracellular pathway patent enough for CLN-5^−^ leukocytes to follow en masse.

In light of the proposed association of endothelial-to-leukocyte transfer of CLN-5 with TEM, it may appear surprising that the MFI for leukocytes in the CNS was not substantially and uniformly greater than that for leukocytes in the blood. Several reasons could contribute. One, it might have some methodological basis, as CNS leukocytes were isolated following collagenase/DNase digestion of brain and spinal cord tissue [68], and errant enzyme activity could have degraded labile, leukocyte-bound CLN-5. Two, junctional proteolysis that accompanies diapedesis [46] might have eliminated some of this CLN-5. Three, the amount of CLN-5 needed for transfer may be subtle, if CLN5^+^ EVs act in a multi-dentate capacity and focally amplify the binding opportunities between leukocytes and endothelial TJs.

Lastly, the disparities in appearance and intensity of CLN-5^+^ cells among the varied leukocyte subtypes could, at least partially, derive from the preference by some to utilize paracellular or transcellular TEM [69–71].

## Conclusions

In summary, this study highlights the time course of appearance of CLN-5^+^ leukocytes in the blood and CNS during the neuroinflammatory, demyelinating condition EAE. Results extend prior work from our group that showed that at least some of this ectopically acquired CLN-5 was transferred from endothelial cells to leukocytes—possibly via EVs released at the BBB. B cells, T cells, monocytes and neutrophils all displayed CLN-5^+^ status during EAE development, though with varying kinetics, absolute numbers, percentages and intensities in both tissue compartments. Higher representations of CLN-5^+^ leukocytes were observed in CNS tissue compared to blood at certain times during disease, and CLN-5^+^ intensity on T cells correlated highly with activation state. These findings are consistent with the interpretation that acquisition of CLN-5 by leukocytes favors their migration across the BBB, possibly by facilitating a zipper mechanism whereby CLN-5 on leukocytes and at endothelial junctions temporarily engage during paracellular diapedesis.

## Supplementary Information

**Additional file 1: Table S1.** Experimental design and immunophenotyping antibodies with their respective fluors.

**Additional file 2: Fig. S1.** Representative plots of CLN-5 expression across different EAE timepoints in CD45^+^ leukocytes. One representative sample was chosen from each experimental group (*n* = 6) based on proximity to the mean value. Density plots of side scatter (SSC-A) vs. CLN-5 staining among CD45^+^ leukocytes in the blood and CNS are shown.

**Additional file 3: Fig. S2.** Representative plots of CLN-5 expression across different EAE timepoints in B cells. One representative sample was chosen from each experimental group (*n* = 6) based on proximity to the mean value. Density plots of side scatter (SSC-A) vs. CLN-5 staining among B cells in the blood and CNS are shown.

**Additional file 4: Fig. S3.** Representative plots of CLN-5 expression across different EAE timepoints in total CD3^+^ T cells and CD4^+^/CD8^+^ subtypes in the blood. One representative sample was chosen from each experimental group (*n* = 6) based on proximity to the mean value. Density plots of side scatter (SSC-A) vs. CLN-5 staining among total CD3^+^ and CD4/8^+^ isolated from the blood are shown.

**Additional file 5: Fig. S4.** Representative plots of CLN-5 expression across different EAE timepoints in total CD3^+^ T cells and CD4^+^/CD8^+^ subtypes in the CNS. One representative sample was chosen from each experimental group (*n* = 6) based on proximity to the mean value. Density plots of side scatter (SSC-A) vs. CLN-5 staining among total CD3^+^ and CD4/8^+^ isolated from the CNS are shown.

**Additional file 6: Fig. S5.** Representative plots of CLN-5 expression across different EAE timepoints in total monocytes and Ly6c^low^ (non-inflammatory)/Ly6C^high^ (inflammatory) subtypes in the blood. One representative sample was chosen from each experimental group (*n* = 6) based on proximity to the mean value. Density plots of side scatter (SSC-A) vs. CLN-5 staining among total monocytes and Ly6c^low^ (non-inflammatory)/Ly6C^high^ (inflammatory) subtypes isolated from the blood are shown.

**Additional file 7: Fig. S6.** Representative plots of CLN-5 expression across different EAE timepoints in total monocytes and Ly6c^low^ (non-inflammatory)/Ly6C^high^ (inflammatory) subtypes in the CNS. One representative sample was chosen from each experimental group (*n* = 6) based on proximity to the mean value. Density plots of side scatter (SSC-A) vs. CLN-5 staining among total monocytes and Ly6c^low^ (non-inflammatory)/Ly6C^high^ (inflammatory) subtypes isolated from the CNS are shown.

**Additional file 8: Fig. S7.** Representative plots of CLN-5 expression across different EAE timepoints in neutrophils. One representative sample was chosen from each experimental group (*n* = 6) based on proximity to the mean value. Density plots of side scatter (SSC-A) vs. CLN-5 staining among neutrophils in the blood and CNS are shown.

**Additional file 9: Fig. S8.** CNS T Cells are nearly all activated. At the indicated days (D) following EAE induction, peripheral blood leukocytes were separately collected from individual mice, and analyzed by flow cytometry. T cells were identified as CD3 positive and CD19 negative. CD44 expression was examined using a pseudocolor plot and CD44^high^ (activated cells) were identified as a distinct population with highest fluorescence. The CD44^high^ gate was created using donor-matched blood and applied to the CNS sample. The % activated T cells is reported, and is ~ 90% at D9–D15. The lesser % activated T cells at D6 is due to the extremely low number of T cells detected in the CNS at this early time. Each experimental group contained six mice. Data are expressed as mean + SEM.

**Additional file 10: Fig. S9.** Correlation between CD44 and CD11c expression by T cells. Peripheral blood leukocytes were collected at D15 following EAE induction, and analyzed by flow cytometry to yield raw fluorescence intensity values for CD44 and CD11c in individual CD3^+^ T cells of each mouse leukocyte sample. Analysis is shown at this particular time, as this was when T cells showed the broadest spectrum of expression of both activation markers. Data were culled from 6 mice at each time-point to yield the regression lines and Pearson Product–Moment Correlation Coefficient (r).

**Additional file 11: Fig. S10.** 3D interpolation of CLN-5, CD44, and CD11c. Peripheral blood leukocytes were collected and analyzed by flow cytometry to yield raw fluorescence intensity values for CLN-5, CD44, and CD11c in individual T cells of each mouse leukocyte sample. Data were culled from 6 mice at D9 following EAE induction (when the highest Pearson r value was obtained for CLN-5 and CD44) to generate a 3D scatter plot of intensity values in *x*, *y*, and *z* planes. Trilinear interpolation is displayed.

**Additional file 12: Fig. S11.** CLN-5 abundance does not correlate highly with inflammatory state of monocytes in blood. Peripheral blood leukocytes were collected at various days (D) following EAE induction, and analyzed by flow cytometry to yield raw fluorescence intensity values for CLN-5 and Ly6C in individual monocytes of each mouse leukocyte sample. Data were culled from 6 mice at each time-point to yield the regression lines and Pearson Product–Moment Correlation Coefficient (r).

**Additional file 13: Fig. S12.** CLN-5 abundance does not correlate highly with activation state of neutrophils in blood. Peripheral blood leukocytes were collected at various days (D) following EAE induction, and analyzed by flow cytometry to yield raw fluorescence intensity values for CLN-5 and CD18 in individual neutrophils of each mouse leukocyte sample. Data were culled from 6 mice at each time-point to yield the regression lines and Pearson Product–Moment Correlation Coefficient (r).

## Data Availability

The data used and/or analyzed during the current study are available from the corresponding author upon reasonable request.

## Abbreviations

BBB: Blood–brain barrier
CLN: Claudin
CNS: Central nervous system
EAE: Experimental autoimmune encephalomyelitis
TJ: Tight junction
