# Supplementary figures and images for: Appearance of claudin-5+ leukocyte subtypes in the blood and CNS during progression of EAE
Source: J Neuroinflammation. 2021 Dec 21;18:296. doi: 10.1186/s12974-021-02328-3 (PMC8691042; doi:10.1186/s12974-021-02328-3)

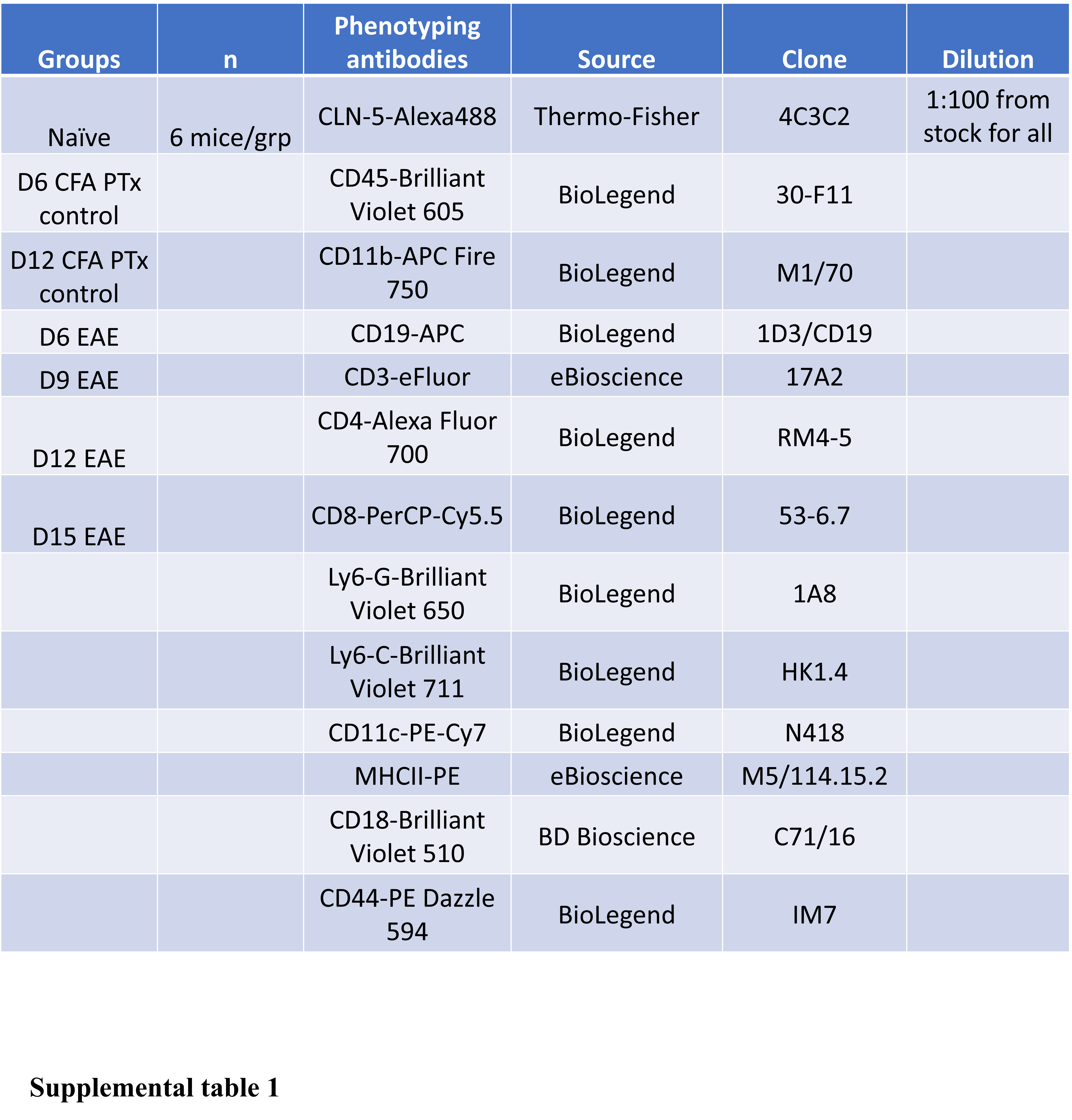

Supplement: Supplementary file 1 — Additional file 1: Table S1. Experimental design and immunophenotyping antibodies with their respective fluors. [file 12974_2021_2328_MOESM1_ESM.tif]

# CD45+ leukocytes

## Blood

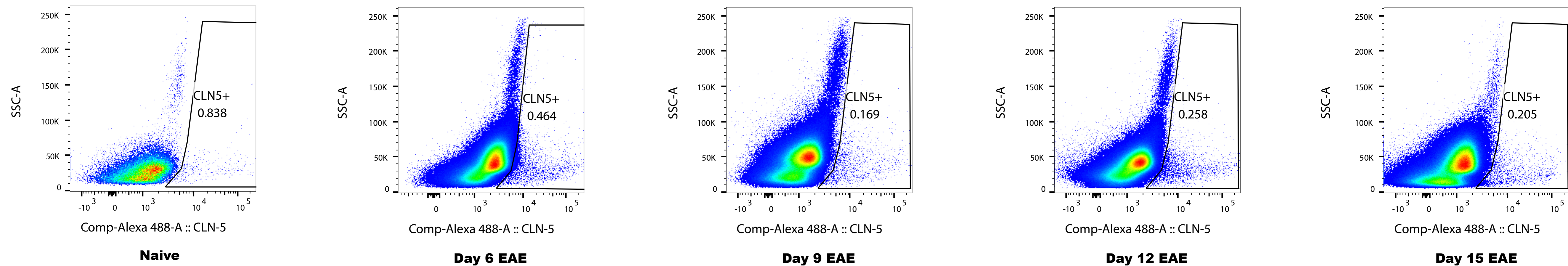

## CNS

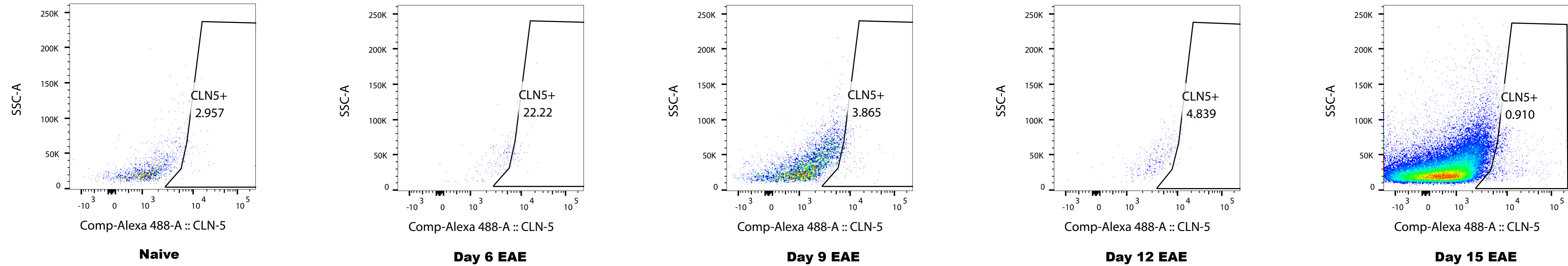

Fig. S1

Supplement: Supplementary file 2 — Additional file 2: Fig. S1. Representative plots of CLN-5 expression across different EAE timepoints in CD45+ leukocytes. One representative sample was chosen from each experimental group (n = 6) based on proximity to the mean value. Density plots of side scatter (SSC-A) vs. CLN-5 staining among CD45+ leukocytes in the blood and CNS are shown. [file 12974_2021_2328_MOESM2_ESM.pdf]

# B cells

## Blood

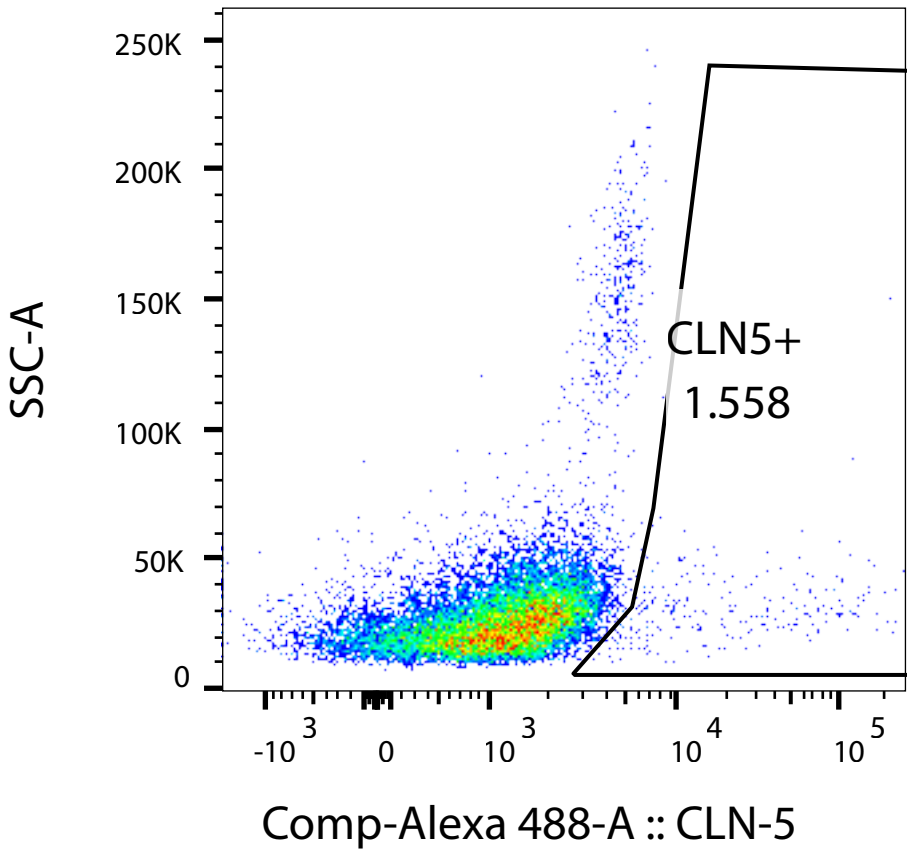

**Naive**

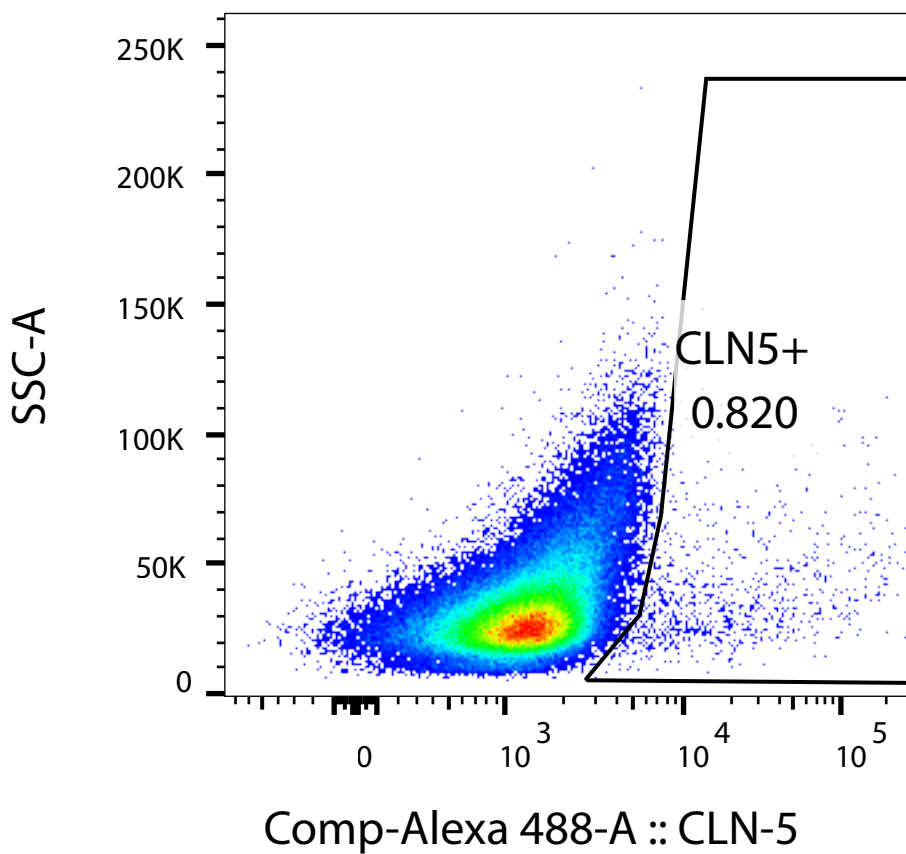

**Day 6 EAE**

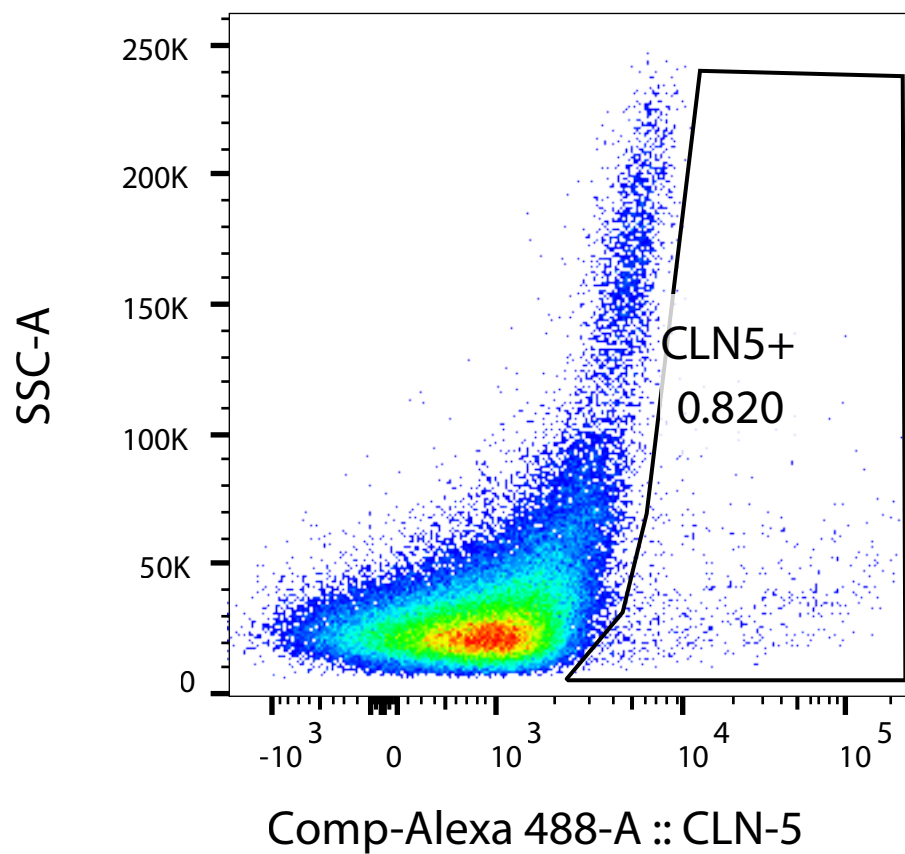

**Day 9 EAE**

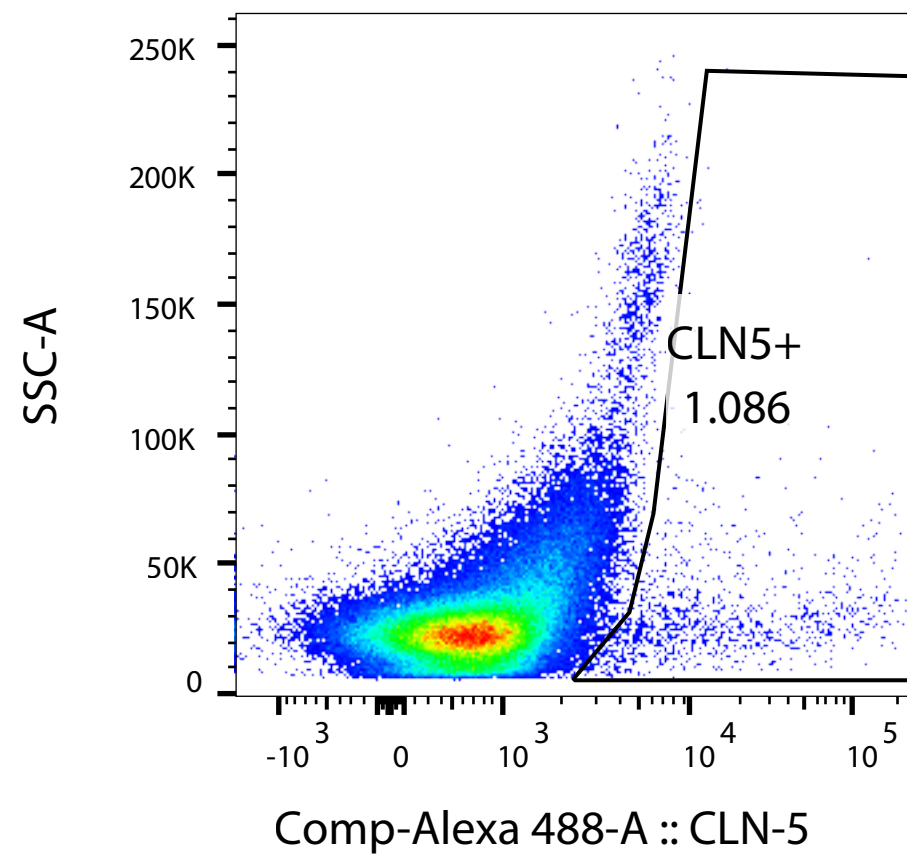

**Day 12 EAE**

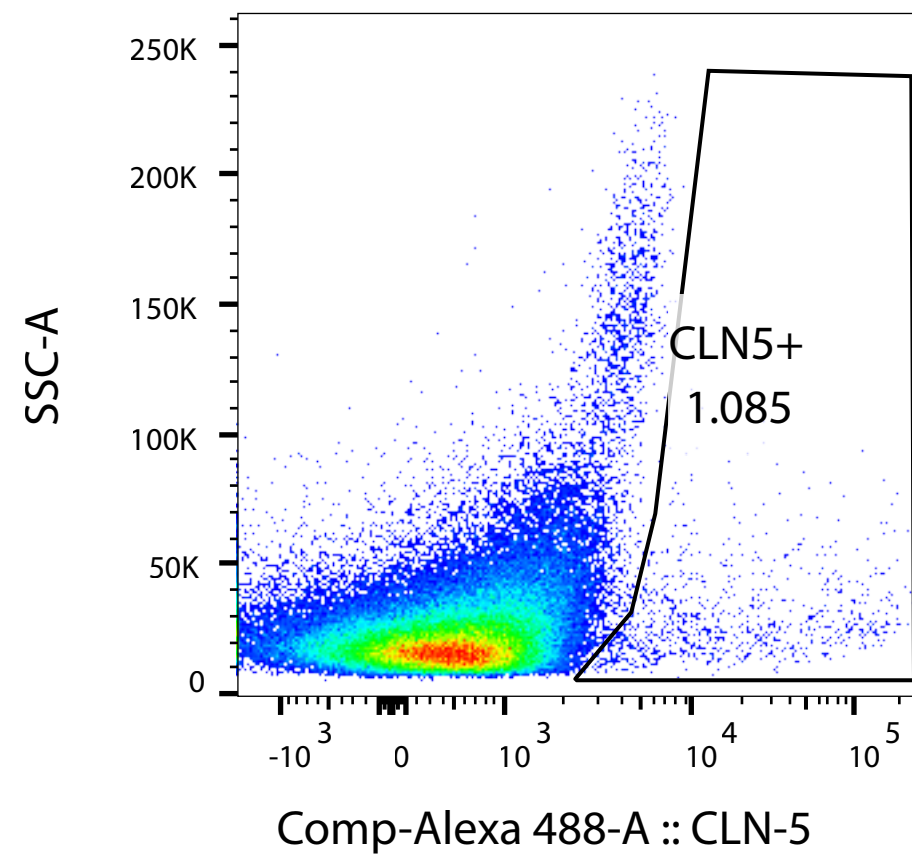

**Day 15 EAE**

## CNS

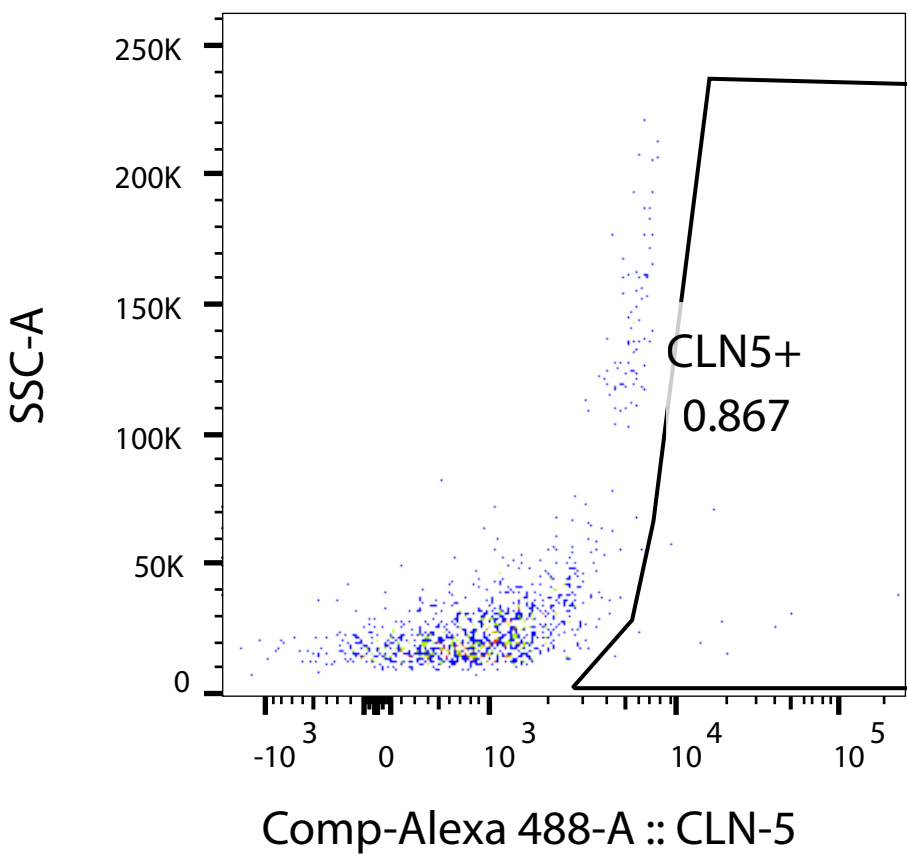

**Naive**

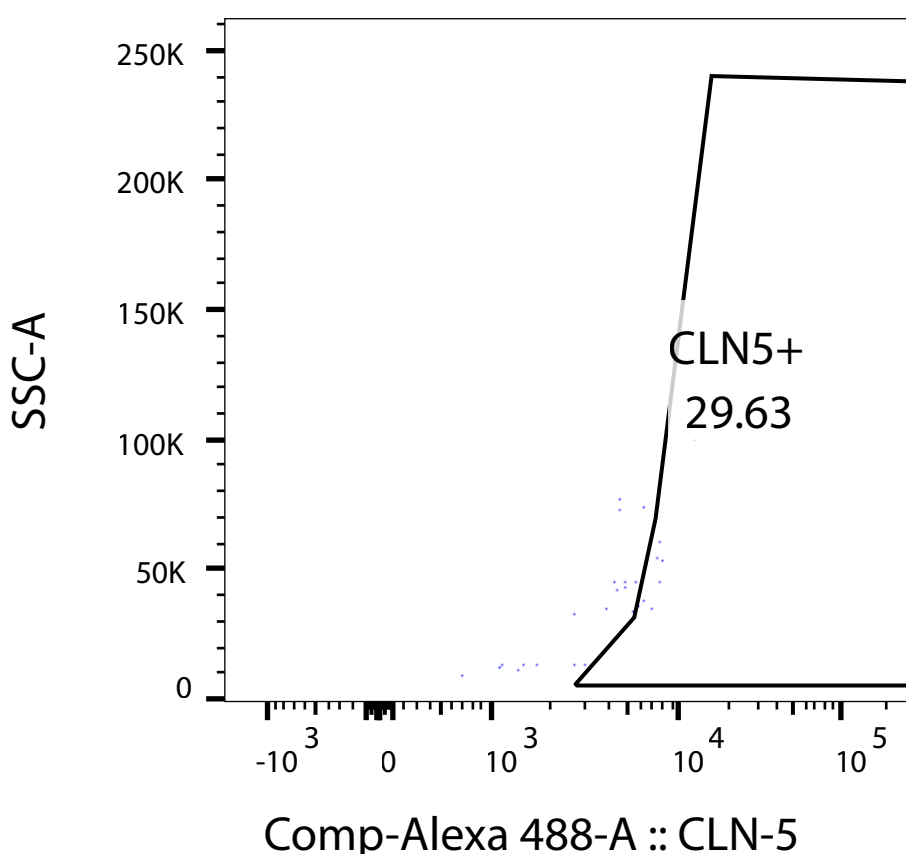

**Day 6 EAE**

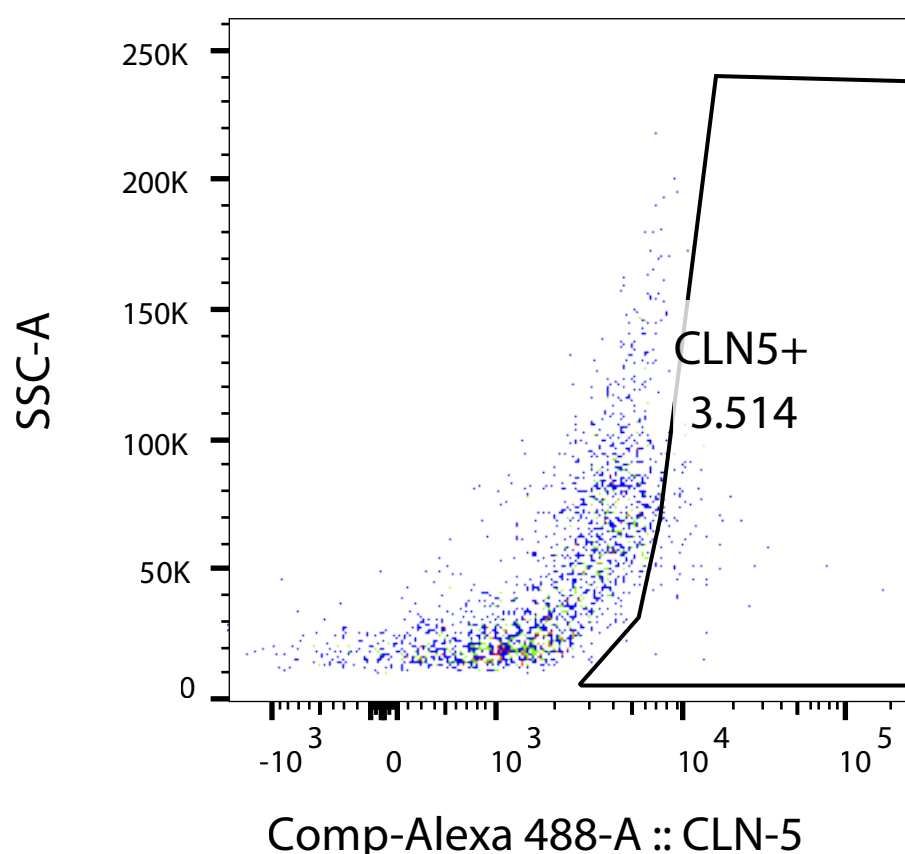

**Day 9 EAE**

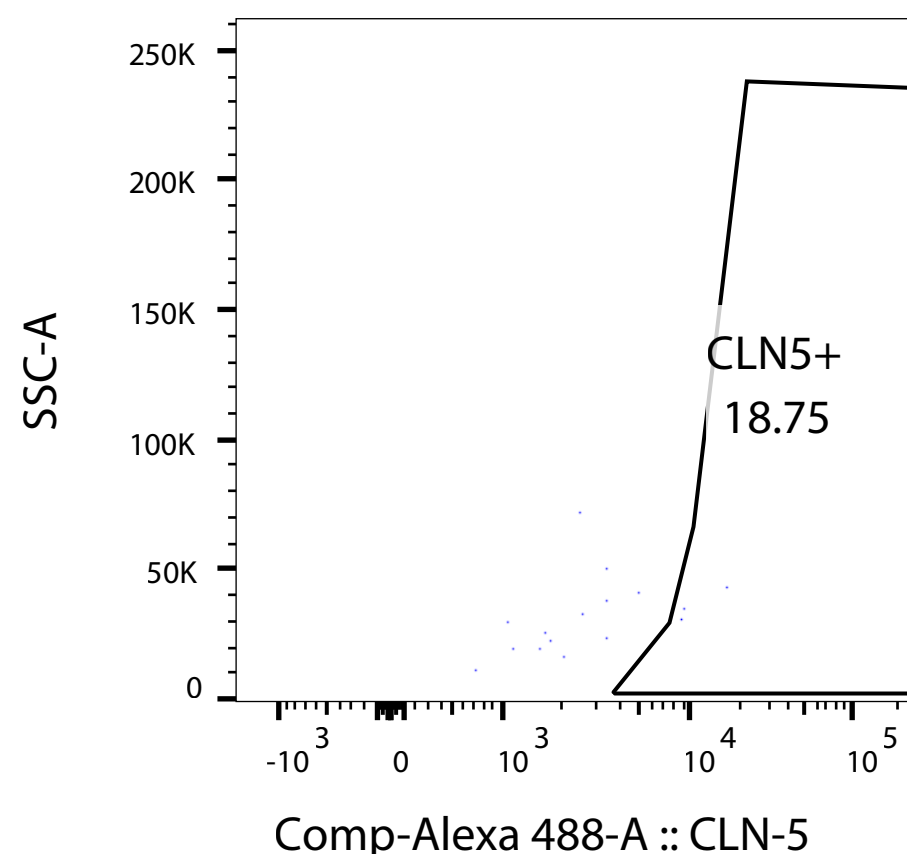

**Day 12 EAE**

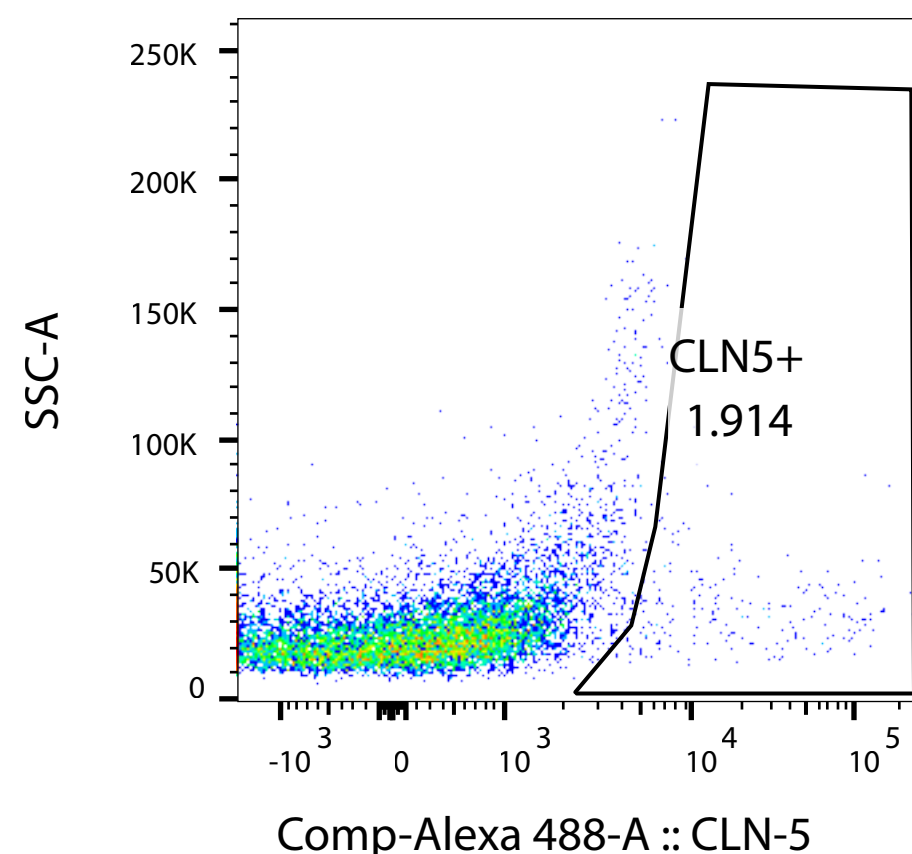

**Day 15 EAE**

Fig. S2

Supplement: Supplementary file 3 — Additional file 3: Fig. S2. Representative plots of CLN-5 expression across different EAE timepoints in B cells. One representative sample was chosen from each experimental group (n = 6) based on proximity to the mean value. Density plots of side scatter (SSC-A) vs. CLN-5 staining among B cells in the blood and CNS are shown. [file 12974_2021_2328_MOESM3_ESM.pdf]

## T cells

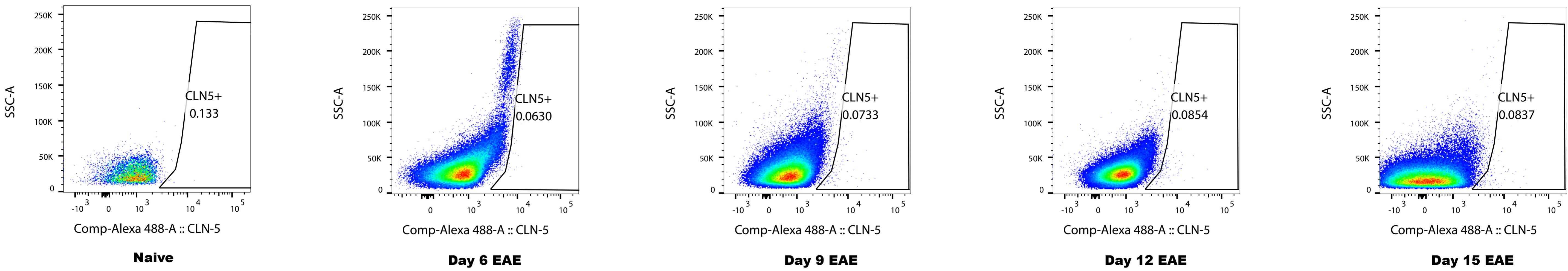

## CD4+ T cells

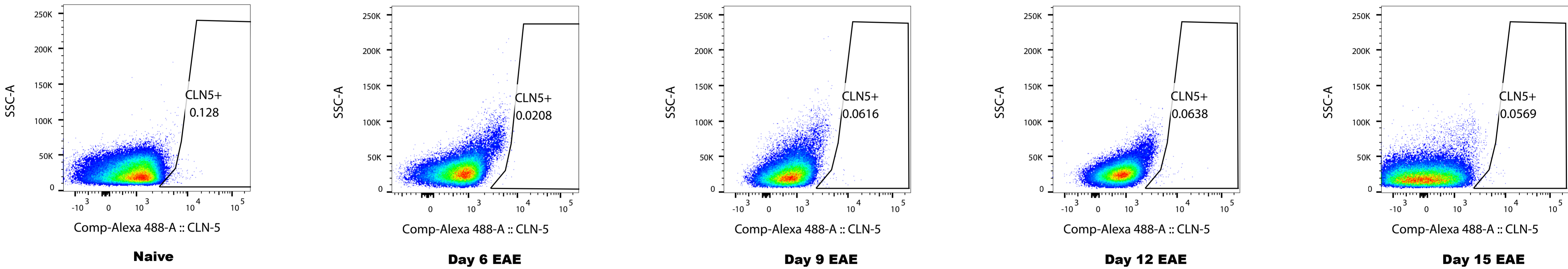

## CD8+ T cells

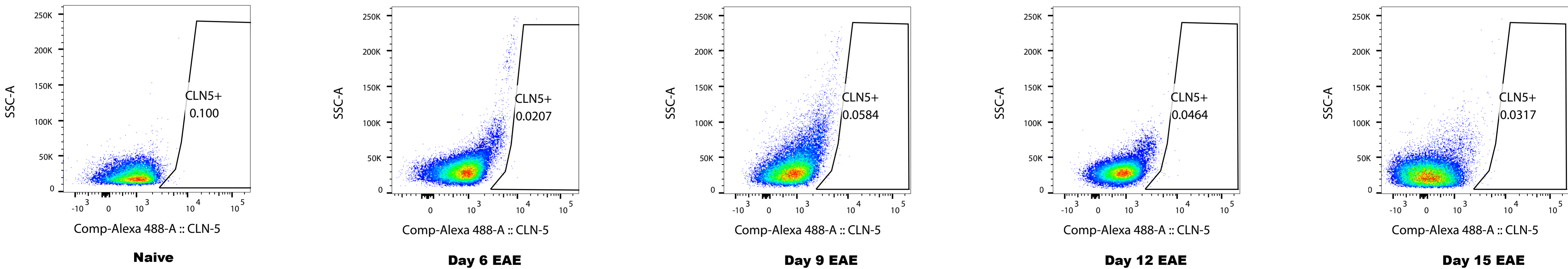

Fig. S3

Supplement: Supplementary file 4 — Additional file 4: Fig. S3. Representative plots of CLN-5 expression across different EAE timepoints in total CD3+ T cells and CD4+/CD8+ subtypes in the blood. One representative sample was chosen from each experimental group (n = 6) based on proximity to the mean value. Density plots of side scatter (SSC-A) vs. CLN-5 staining among total CD3+ and CD4/8+ isolated from the blood are shown. [file 12974_2021_2328_MOESM4_ESM.pdf]

## T cells

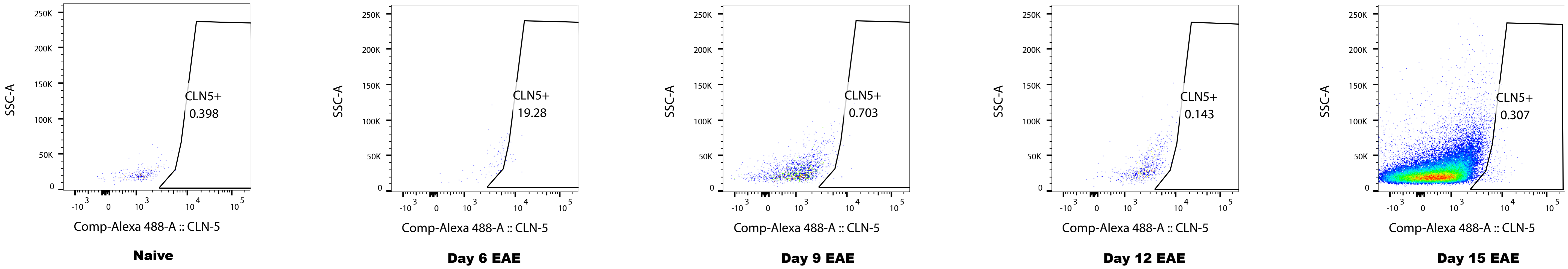

## CD4+ T cells

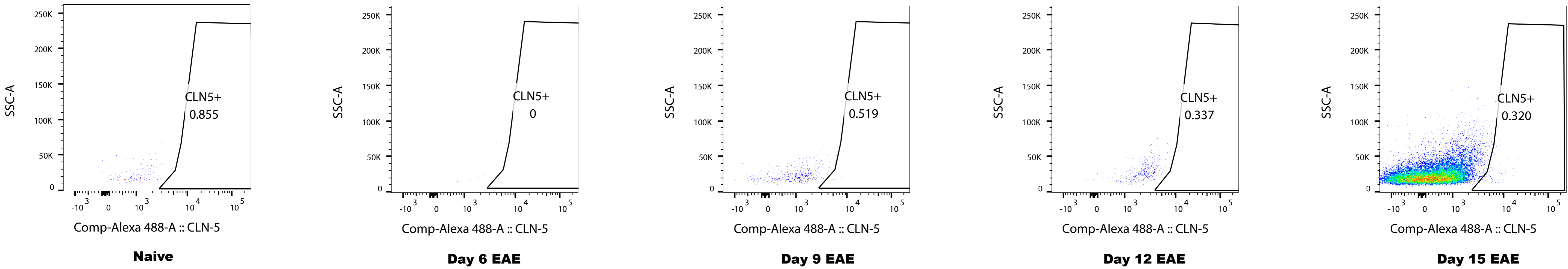

## CD8+ T cells

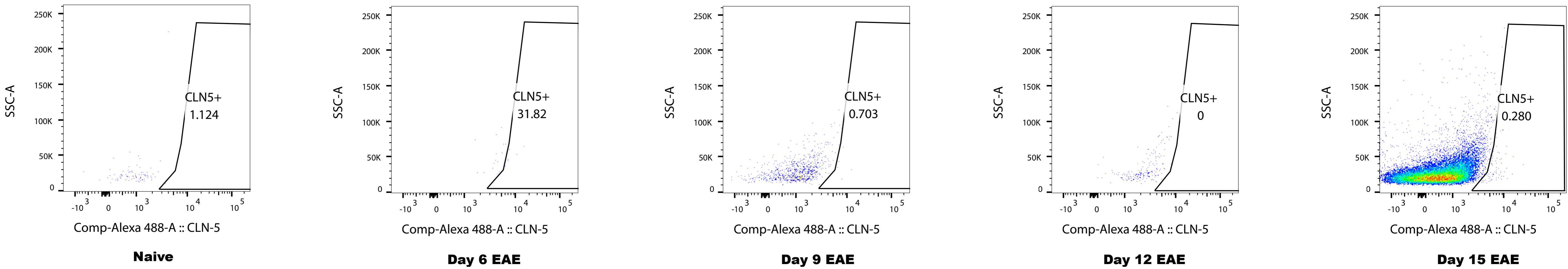

Fig. S4

Supplement: Supplementary file 5 — Additional file 5: Fig. S4. Representative plots of CLN-5 expression across different EAE timepoints in total CD3+ T cells and CD4+/CD8+ subtypes in the CNS. One representative sample was chosen from each experimental group (n = 6) based on proximity to the mean value. Density plots of side scatter (SSC-A) vs. CLN-5 staining among total CD3+ and CD4/8+ isolated from the CNS are shown. [file 12974_2021_2328_MOESM5_ESM.pdf]

## Monocytes

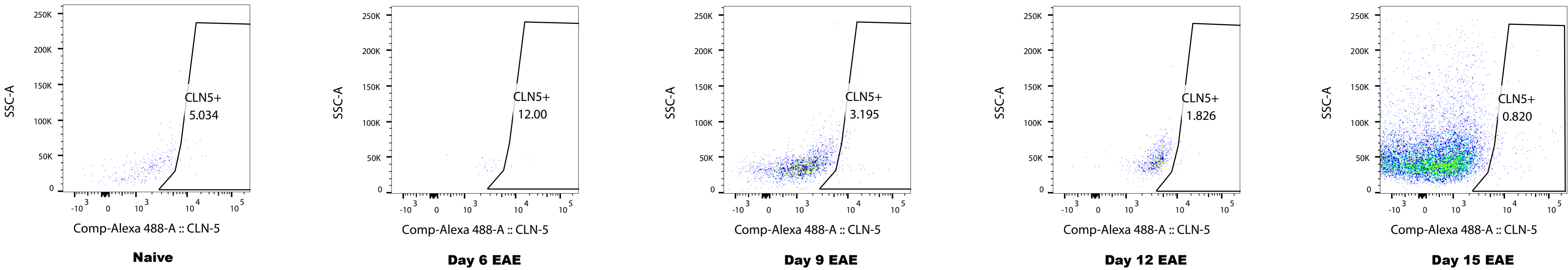

## Non-inflammatory monocytes

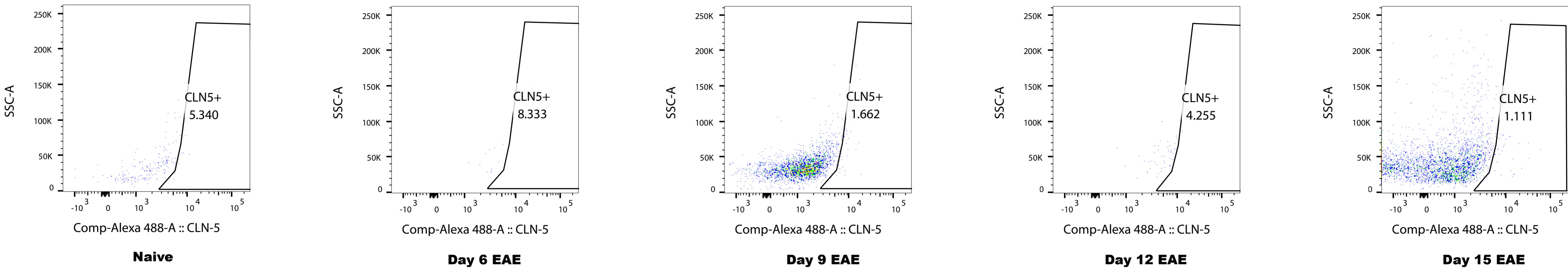

## Inflammatory monocytes

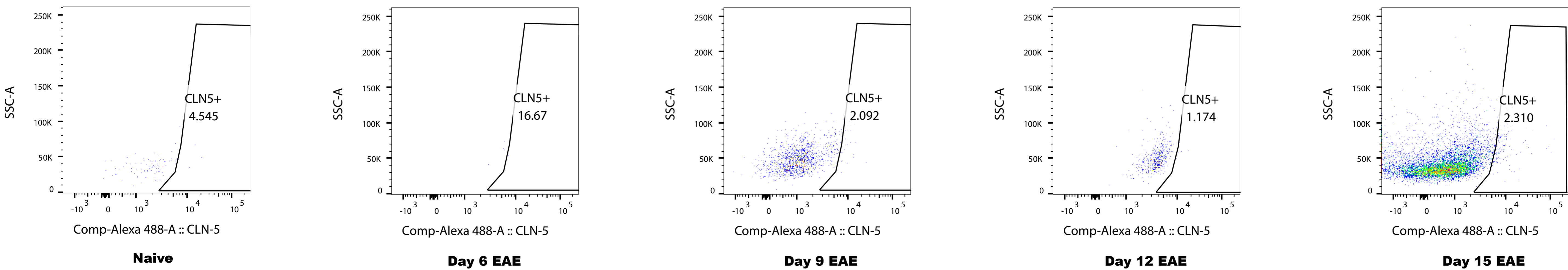

Fig. S6

Supplement: Supplementary file 7 — Additional file 7: Fig. S6. Representative plots of CLN-5 expression across different EAE timepoints in total monocytes and Ly6clow (non-inflammatory)/Ly6Chigh (inflammatory) subtypes in the CNS. One representative sample was chosen from each experimental group (n = 6) based on proximity to the mean value. Density plots of side scatter (SSC-A) vs. CLN-5 staining among total monocytes and Ly6clow (non-inflammatory)/Ly6Chigh (inflammatory) subtypes isolated from the CNS are shown. [file 12974_2021_2328_MOESM7_ESM.pdf]

# Neutrophils

## Blood

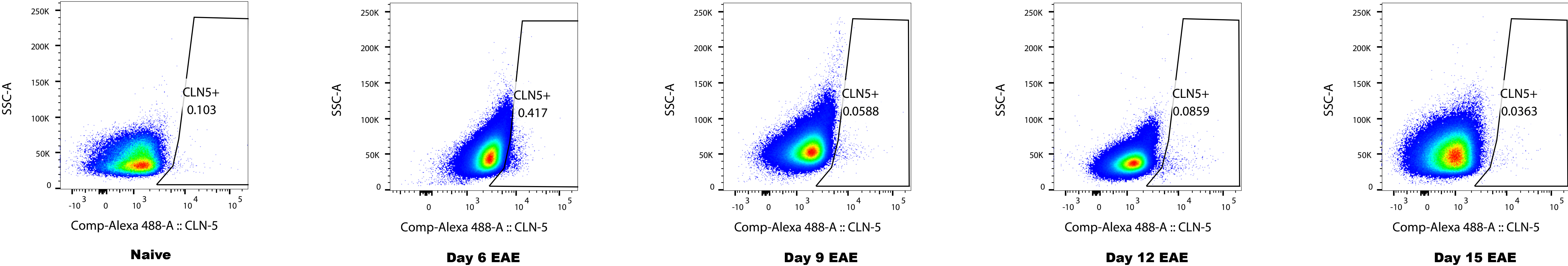

## CNS

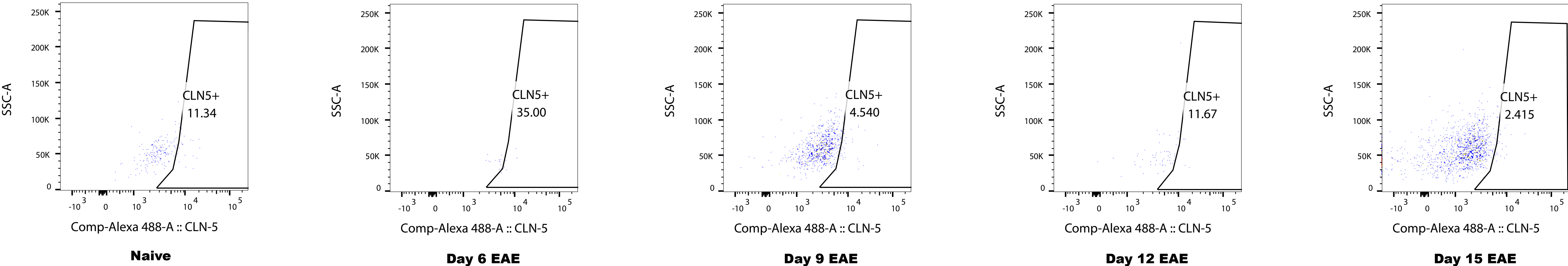

Fig. S7

Supplement: Supplementary file 8 — Additional file 8: Fig. S7. Representative plots of CLN-5 expression across different EAE timepoints in neutrophils. One representative sample was chosen from each experimental group (n = 6) based on proximity to the mean value. Density plots of side scatter (SSC-A) vs. CLN-5 staining among neutrophils in the blood and CNS are shown. [file 12974_2021_2328_MOESM8_ESM.pdf]

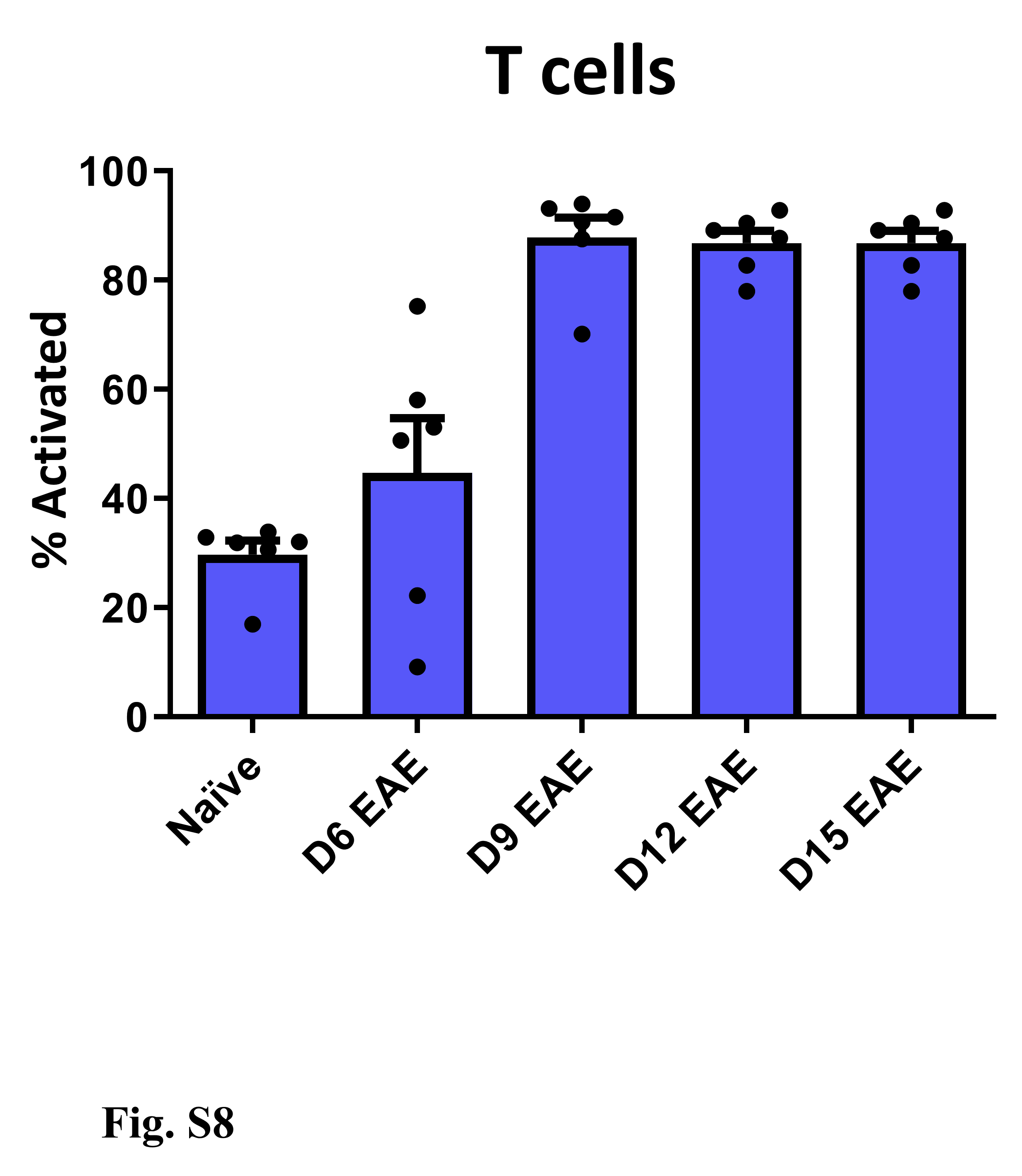

Supplement: Supplementary file 9 — Additional file 9: Fig. S8. CNS T Cells are nearly all activated. At the indicated days (D) following EAE induction, peripheral blood leukocytes were separately collected from individual mice, and analyzed by flow cytometry. T cells were identified as CD3 positive and CD19 negative. CD44 expression was examined using a pseudocolor plot and CD44high (activated cells) were identified as a distinct population with highest fluorescence. The CD44high gate was created using donor-matched blood and applied to the CNS sample. The % activated T cells is reported, and is ~ 90% at D9–D15. The lesser % activated T cells at D6 is due to the extremely low number of T cells detected in the CNS at this early time. Each experimental group contained six mice. Data are expressed as mean + SEM. [file 12974_2021_2328_MOESM9_ESM.tif]

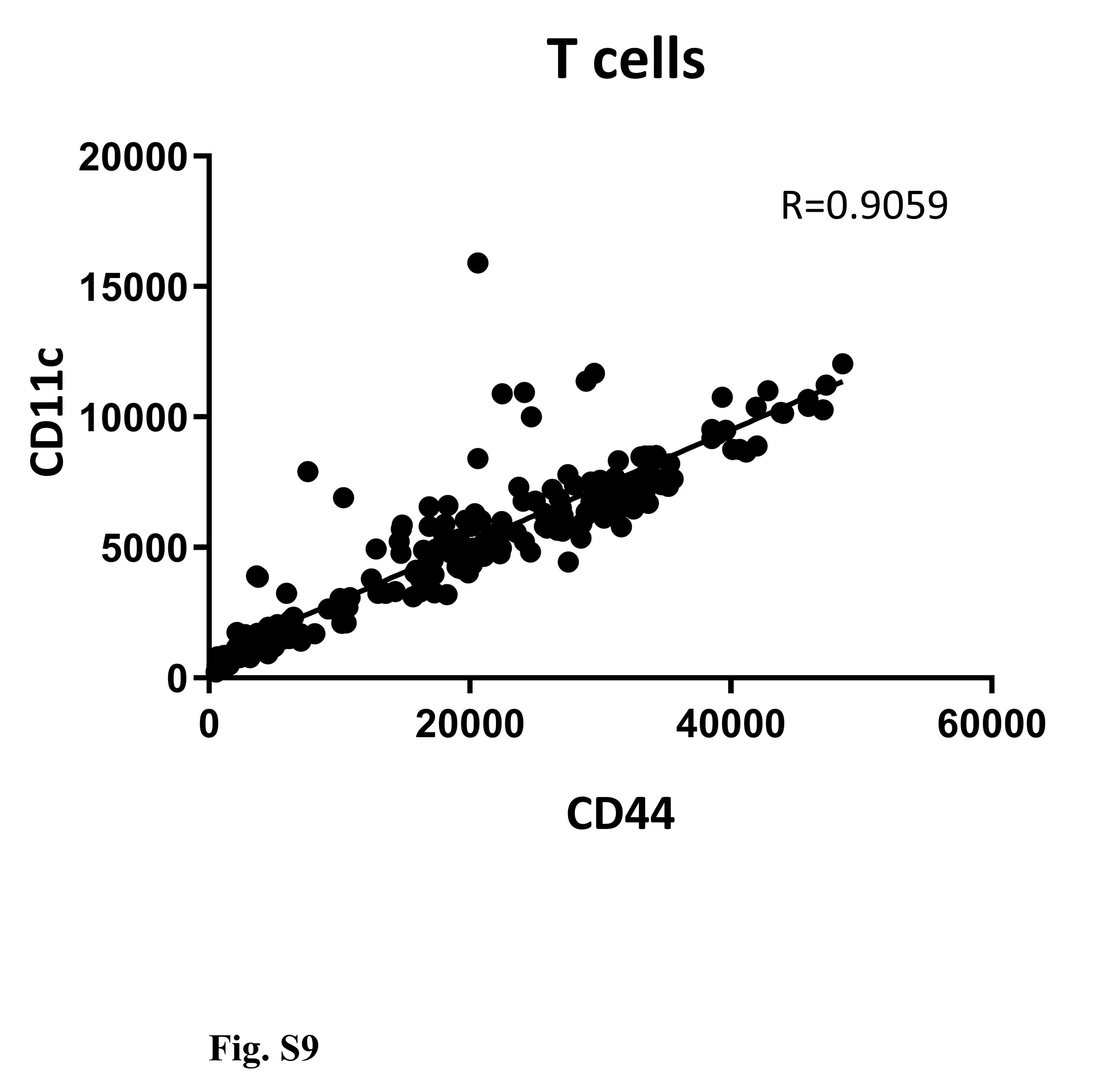

Supplement: Supplementary file 10 — Additional file 10: Fig. S9. Correlation between CD44 and CD11c expression by T cells. Peripheral blood leukocytes were collected at D15 following EAE induction, and analyzed by flow cytometry to yield raw fluorescence intensity values for CD44 and CD11c in individual CD3+ T cells of each mouse leukocyte sample. Analysis is shown at this particular time, as this was when T cells showed the broadest spectrum of expression of both activation markers. Data were culled from 6 mice at each time-point to yield the regression lines and Pearson Product–Moment Correlation Coefficient (r). [file 12974_2021_2328_MOESM10_ESM.tif]

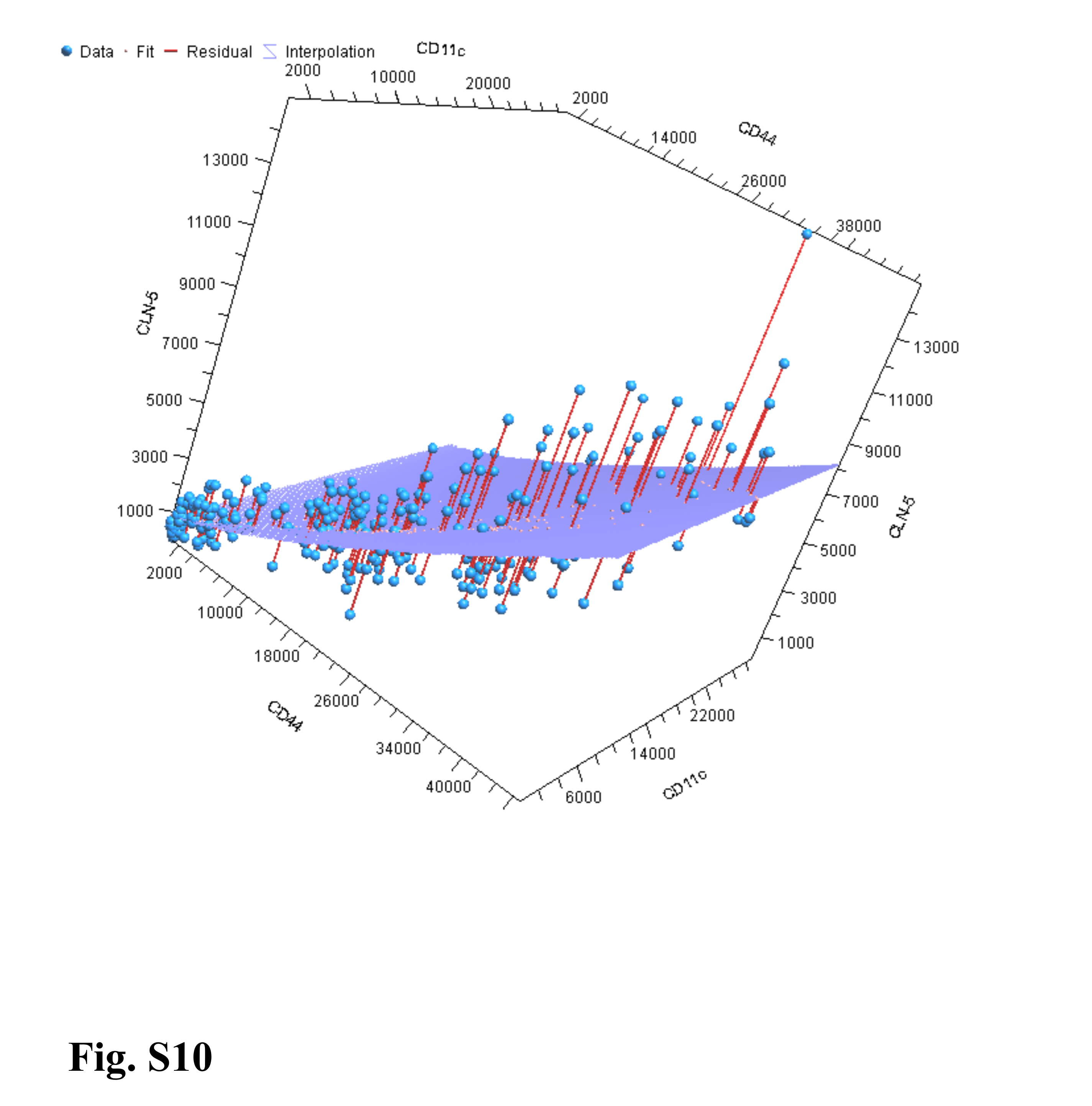

Supplement: Supplementary file 11 — Additional file 11: Fig. S10. 3D interpolation of CLN-5, CD44, and CD11c. Peripheral blood leukocytes were collected and analyzed by flow cytometry to yield raw fluorescence intensity values for CLN-5, CD44, and CD11c in individual T cells of each mouse leukocyte sample. Data were culled from 6 mice at D9 following EAE induction (when the highest Pearson r value was obtained for CLN-5 and CD44) to generate a 3D scatter plot of intensity values in x, y, and z planes. Trilinear interpolation is displayed. [file 12974_2021_2328_MOESM11_ESM.tif]

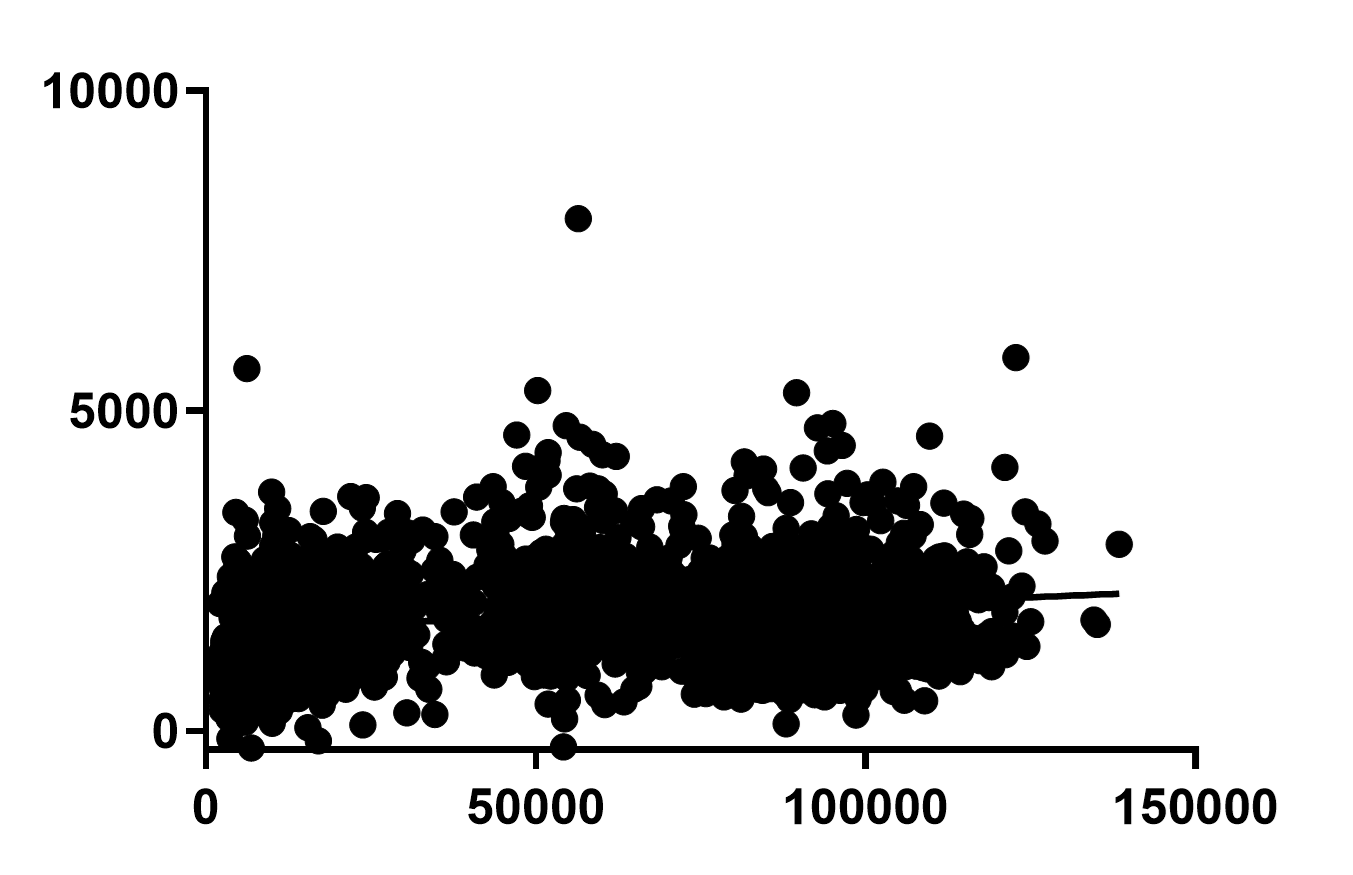

Supplement: Supplementary file 12 — Additional file 12: Fig. S11. CLN-5 abundance does not correlate highly with inflammatory state of monocytes in blood. Peripheral blood leukocytes were collected at various days (D) following EAE induction, and analyzed by flow cytometry to yield raw fluorescence intensity values for CLN-5 and Ly6C in individual monocytes of each mouse leukocyte sample. Data were culled from 6 mice at each time-point to yield the regression lines and Pearson Product–Moment Correlation Coefficient (r). [file 12974_2021_2328_MOESM12_ESM.tif]

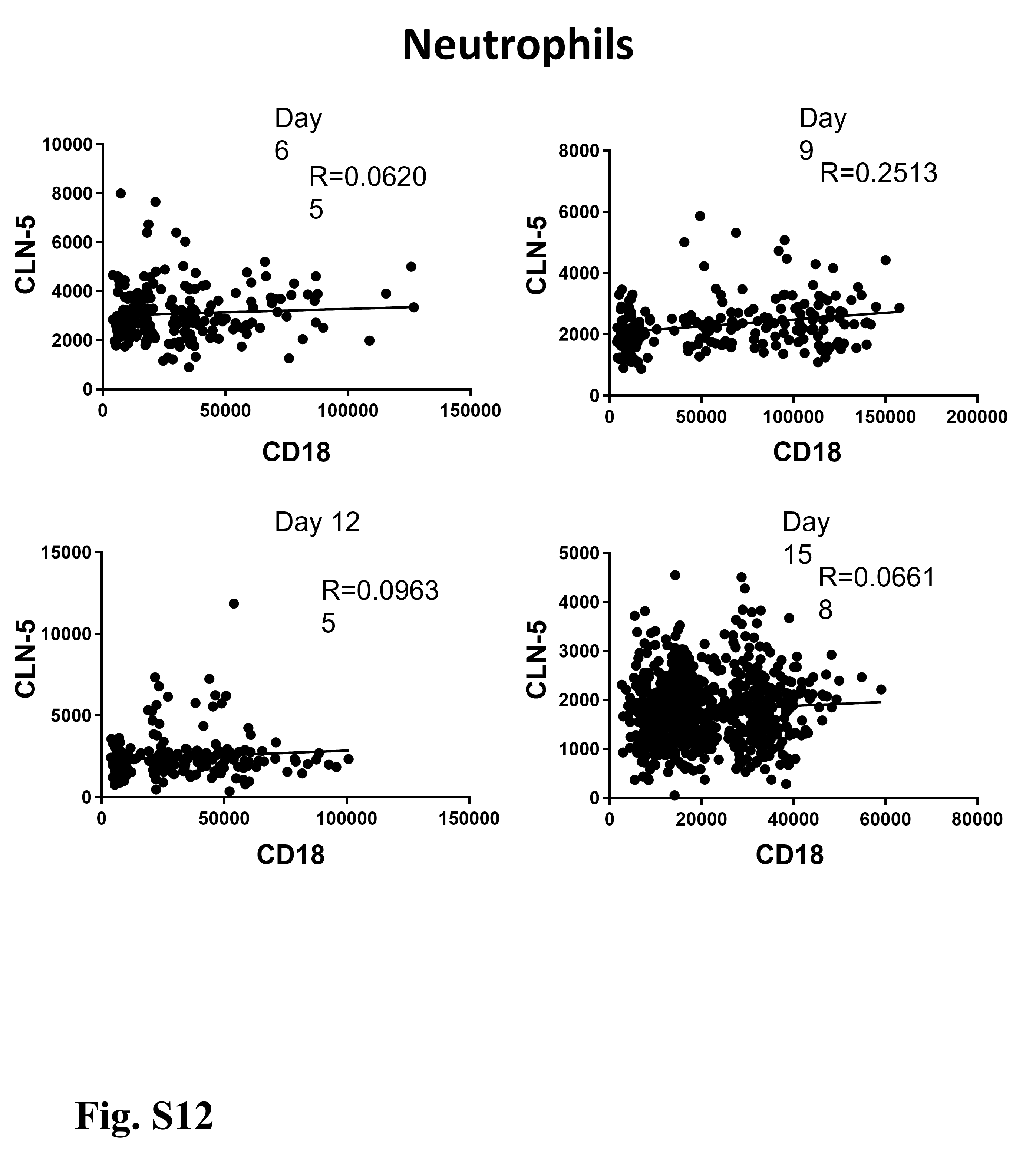

Supplement: Supplementary file 13 — Additional file 13: Fig. S12. CLN-5 abundance does not correlate highly with activation state of neutrophils in blood. Peripheral blood leukocytes were collected at various days (D) following EAE induction, and analyzed by flow cytometry to yield raw fluorescence intensity values for CLN-5 and CD18 in individual neutrophils of each mouse leukocyte sample. Data were culled from 6 mice at each time-point to yield the regression lines and Pearson Product–Moment Correlation Coefficient (r). [file 12974_2021_2328_MOESM13_ESM.tif]
