# Supplementary material for: Appearance of claudin-5+ leukocyte subtypes in the blood and CNS during progression of EAE
Source: J Neuroinflammation. 2021 Dec 21;18:296. doi: 10.1186/s12974-021-02328-3 (PMC8691042; doi:10.1186/s12974-021-02328-3)
Supplement: Supplementary file 6 — Additional file 6: Fig. S5. Representative plots of CLN-5 expression across different EAE timepoints in total monocytes and Ly6clow (non-inflammatory)/Ly6Chigh (inflammatory) subtypes in the blood. One representative sample was chosen from each experimental group (n = 6) based on proximity to the mean value. Density plots of side scatter (SSC-A) vs. CLN-5 staining among total monocytes and Ly6clow (non-inflammatory)/Ly6Chigh (inflammatory) subtypes isolated from the blood are shown. [file 12974_2021_2328_MOESM6_ESM.pdf]

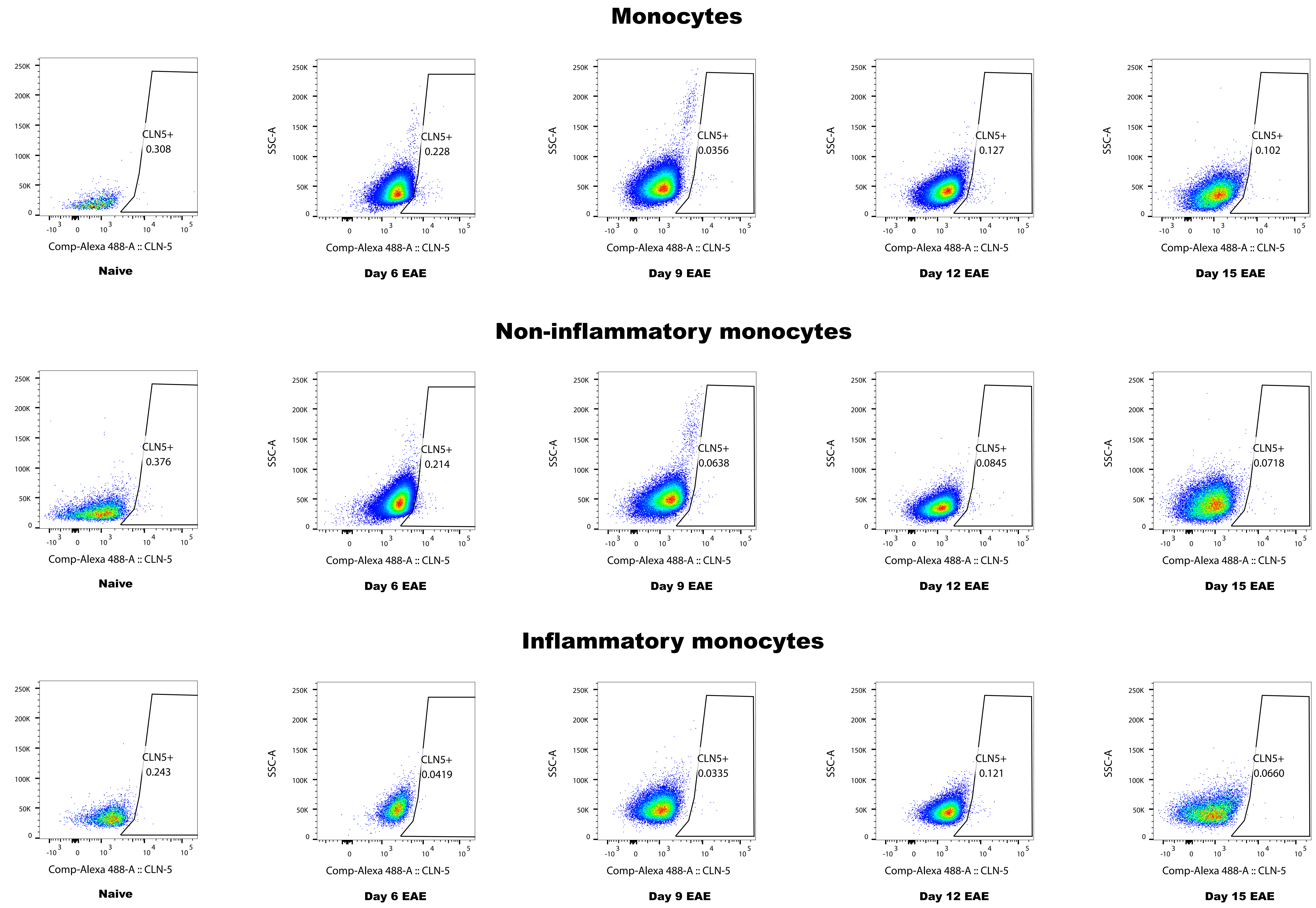
Monocytes
CLN5+ 0.308
CLN5+ 0.228
CLN5+ 0.0356
CLN5+ 0.127
CLN5+ 0.102
SSC-A
Comp-Alexa 488-A :: CLN-5
Naive
Day 6 EAE
Day 9 EAE
Day 12 EAE
Day 15 EAE
Non-inflammatory monocytes
CLN5+ 0.376
CLN5+ 0.214
CLN5+ 0.0638
CLN5+ 0.0845
CLN5+ 0.0718
Inflammatory monocytes
CLN5+ 0.243
CLN5+ 0.0419
CLN5+ 0.0335
CLN5+ 0.121
CLN5+ 0.0660

Fig. S5
